# Genomic Insights into the Increased Occurrence of Campylobacteriosis Caused by Antimicrobial-Resistant Campylobacter coli

**DOI:** 10.1128/mbio.02835-22

**Published:** 2022-12-06

**Authors:** Penghang Zhang, Xiaoai Zhang, Yuzhu Liu, Qingpo Cui, Xiaoxia Qin, Yanlin Niu, Chao Wang, TongYu Wang, Qian Chen, Shuangyang Ding, Xiaochen Ma, Zhangqi Shen

**Affiliations:** a Beijing Key Laboratory of Detection Technology for Animal-Derived Food Safety, College of Veterinary Medicine, China Agricultural University, Beijing, China; b Institute for Nutrition and Food Hygiene, Beijing Center for Disease Prevention and Control, Beijing, China; c Department of Preventive Veterinary Medicine, College of Veterinary Medicine, China Agricultural University, Beijing, China; University of Pittsburgh

**Keywords:** *Campylobacter coli*, whole-genome sequencing, core genome multilocus sequence typing, antimicrobial resistance

## Abstract

Campylobacter is the leading bacterial cause of diarrheal illnesses worldwide. Campylobacter jejuni and C. coli are the most common species accounting for campylobacteriosis. Although the proportion of campylobacteriosis caused by C. coli is increasing rapidly in China, the underlying mechanisms of this emergence remain unclear. In this study, we analyzed the whole-genome sequences and associated environments of 1,195 C. coli isolates with human, poultry, or porcine origins from 1980 to 2021. C. coli isolates of human origin were closely related to those from poultry, suggesting that poultry was the main source of C. coli infection in humans. Analysis of antimicrobial resistance determinants indicated that the prevalence of multidrug-resistant C. coli has increased dramatically since the 2010s, coinciding with the shift in abundance from C. jejuni to C. coli in Chinese poultry. Compared with C. jejuni, drug-resistant C. coli strains were better adapted and showed increased proliferation in the poultry production environment, where multiple antimicrobial agents were frequently used. This study provides an empirical basis for the molecular mechanisms that have enabled C. coli to become the dominant Campylobacter species in poultry; we also emphasize the importance of poultry products as sources of campylobacteriosis caused by C. coli in human patients.

## INTRODUCTION

Campylobacter species, especially Campylobacter jejuni and Campylobacter coli, are major foodborne pathogens and the leading bacterial cause of gastroenteritis in humans worldwide (1–3). The World Health Organization has listed Campylobacter as one of the four key global causes of diarrheal diseases (4); in the European Union, the most reported zoonotic diseases in humans in 2019 were due to Campylobacter (5); and in the United States, the Foodborne Diseases Active Surveillance Network (FoodNet) reported in 2021 that approximately 20 cases per 100,000 people were diagnosed each year. Nevertheless, many cases go undiagnosed or unreported (6). Most Campylobacter infections are self-limiting, with antimicrobial treatment, particularly fluoroquinolones and macrolides, being necessary only in severe or prolonged cases (7, 8). In the past few decades, antimicrobial agents, including macrolides, florfenicol, fluoroquinolones, and tetracyclines, were extensively used in the poultry and livestock industries, resulting in the rapid emergence of multidrug-resistant (MDR) Campylobacter in both humans and animals (9). MDR Campylobacter poses great clinical challenges (10). In 2017, the World Health Organization listed Campylobacter that is resistant to fluoroquinolone as one of six high-priority antimicrobial-resistant (AMR) pathogens (11). The overall resistance rates of Campylobacter to macrolides were relatively low in the United States during 2015 to 2021. C. coli generally has higher rates of resistance to macrolides than C. jejuni (0.7 to 12.4% versus 0.0 to 1.9%) (12). In China, C. coli from chicken products showed extremely high rates of resistance to macrolides (from 73% to 100%) (13–15), which would inhibit the treatment of campylobacteriosis caused by C. coli.

Most cases of human campylobacteriosis are attributed to C. jejuni, whereas C. coli contributes to around 5% of cases (16–18). In recent years, the proportion of campylobacteriosis caused by C. coli has increased to 15% in China (19). In China from 2008 to 2014, C. coli gradually replaced C. jejuni as the dominant Campylobacter species in poultry from five provinces (15), though the underlying mechanisms of this shift remain unclear.

In the present study, we analyzed the whole-genome sequences of 1,195 C. coli isolates from human, poultry, swine, and their associated environments. Data from 1980 to 2021 were combined from our lab and GenBank. Our study showed that the acquisition of MDR in C. coli isolates could have facilitated their emergence in the Chinese poultry industry, where multiple antimicrobial agents were frequently used, in turn promoting the occurrence of campylobacteriosis caused by C. coli.

## RESULTS

### Outcome of bacterial isolation.

In total, 11,125 stool samples were collected from individual diarrhea patients in 19 hospitals in Beijing during 2016 and 2021. Overall, 690 Campylobacter isolates were recovered, including 586 isolates of C. jejuni (84.93% [586/690]) and 104 isolates of C. coli (15.07% [104/690]). Coinfections were found in seven cases, of which six were due to coinfection with C. jejuni and C. coli and one was due to coinfection with two distinct C. jejuni isolates (see Table S1 in the supplemental material). In addition, we isolated Campylobacter strains from 158 samples of poultry (broilers and ducks) origin. In total, 29 C. jejuni and 31 C. coli isolates were obtained. Another 8 C. coli isolates were obtained from swine samples and other animal samples (Table S1). We found that C. coli is a dominant Campylobacter species isolated from swine; however, the prevalence of C. coli from swine meat was much lower than that from poultry meat.

10.1128/mbio.02835-22.6 TABLE S1 Prevalence of Campylobacter isolates detected in patients and animal meats in Beijing. Download Table S1, DOCX file, 0.01 MB. Copyright © 2022 Zhang et al. 2022 Zhang et al. https://creativecommons.org/licenses/by/4.0/ This content is distributed under the terms of the Creative Commons Attribution 4.0 International license.

### Total whole-genome sequences.

We analyzed the whole-genome sequences (WGSs) of 1,195 C. coli isolates from 1980 to 2021: 141 were newly isolated and sequenced, while the remaining 1,054 were downloaded from GenBank (downloaded on 13 November 2021). The isolates originated from humans (*n* = 581), poultry and the associated environment (*n* = 265), dairy cattle and the associated environment (*n* = 142), swine (*n* = 36), the natural environment (*n* = 114), and other animals (*n* = 57) (Table S2). The geographical sources include the United Kingdom (*n* = 598), the United States (*n* = 186), China (*n* = 185), and 22 other countries/regions (*n* = 226). In addition, 498 genomic sequences of Campylobacter jejuni in GenBank were added for phylogenetic analysis. Detailed information on the isolates in GenBank is shown in Table S3.

10.1128/mbio.02835-22.7 TABLE S2 Summary of C. coli isolates recovered in this study and downloaded from GenBank. Download Table S2, DOCX file, 0.01 MB. Copyright © 2022 Zhang et al. 2022 Zhang et al. https://creativecommons.org/licenses/by/4.0/ This content is distributed under the terms of the Creative Commons Attribution 4.0 International license.

10.1128/mbio.02835-22.8 TABLE S3 Geographic locations and source information of C. coli and C. jejuni isolates in GenBank. Download Table S3, DOCX file, 0.02 MB. Copyright © 2022 Zhang et al. 2022 Zhang et al. https://creativecommons.org/licenses/by/4.0/ This content is distributed under the terms of the Creative Commons Attribution 4.0 International license.

### C. coli isolates from human and poultry were phylogenetically related.

**FIG 1:**
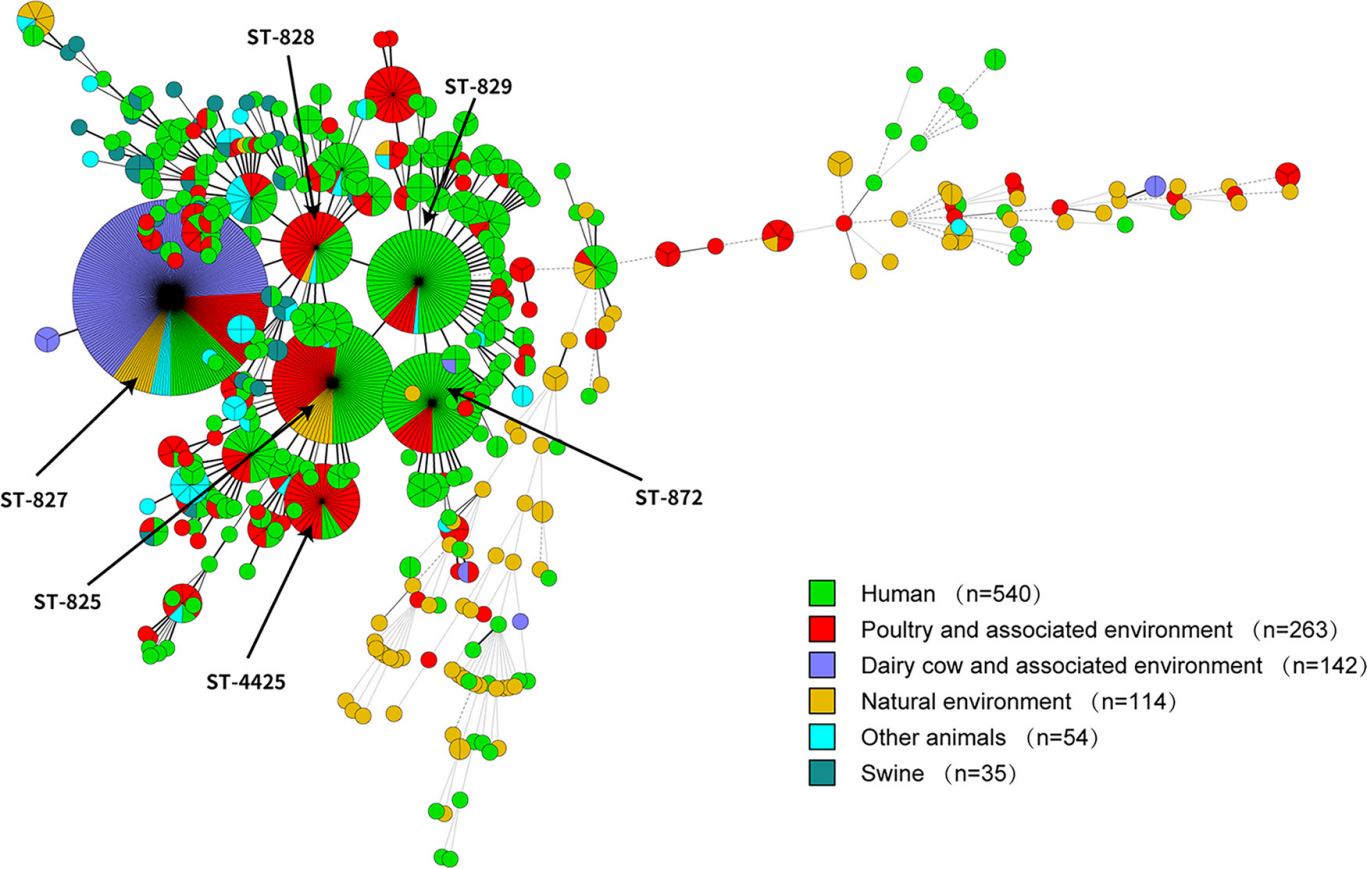
Phylogenetic analysis. Minimum spanning tree of 1,148 C. coli isolates from GenBank and our lab. Isolates are represented by circles, and the size of the circle is proportional to the number of isolates. The sources of the isolates are indicated in the key.

Multilocus sequence typing identified 365 distinct sequence types (STs) in 1,148 of 1,195 isolates, and 42 different multilocus sequence typing (MLST) locus combinations were identified in the remaining 47 isolates (Fig. 1). From 540 human-derived isolates, 209 STs were identified, with the most commonly isolated ST being ST-829 (52/540 [9.63%]), followed by ST-872 (47/540 [8.70%]), ST-825 (39/540 [7.22%]), and ST-827 (27/540 [5.00%]). ST-825 (31/263 [11.79%]) was also the most common ST in poultry and the associated environment, followed by ST-4425 (28/263 [10.65%]) and ST-827 (27/263 [10.27%]). In contrast, the most commonly isolated STs from swine and other animals were ST-900 (3/35 [8.57%]) and ST-9493 (8/54 [14.81%]), while ST-900 was found only in one human isolate. It is worth noting that the STs of other isolates were scattered, that there were only six STs in dairy cattle and the associated environment, and that ST-827 was identified in 94.37% (134/142) of isolates. This might be because 97.18% (138/142) of isolates from dairy cattle and the associated environment were collected in the United Kingdom in 2014.

**FIG 2:**
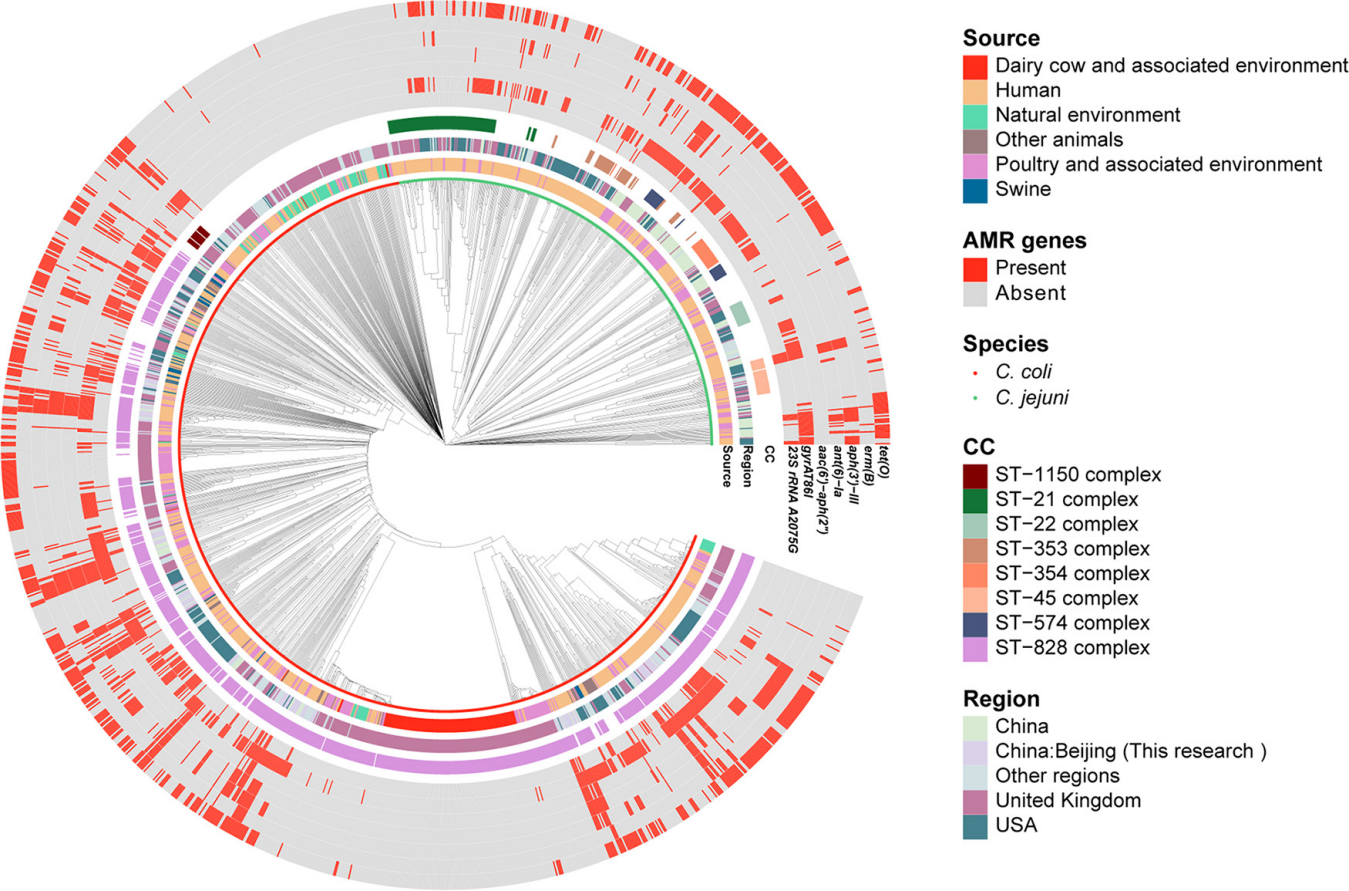
Comparison of Campylobacter isolates based on a single-linkage analysis of the cgMLST profiles from GenBank and our lab.

Core genome multilocus sequence typing (cgMLST) analysis showed a long genetic distance between C. jejuni and C. coli regardless of the source and region of isolates and revealed a close genetic relationship among C. coli isolates from human and poultry and their associated environments. In total, 460 cgMLST_200_ groups were formed for 1,195 isolates, and 325 cgMLST_200_ groups contained only one isolate (Fig. 2). Among cgMLST_200_ groups containing more than two isolates, 62.96% (85/135) contained isolates from a single source. In cgMLST_200_ groups containing isolates from more than two sources, 66.00% (33/50) were from human and poultry sources, which was much higher than other sources combined. In addition, 54.71% (145/265) of isolates from poultry and the associated environment occurred in the same cgMLST_200_ groups as human isolates. Meanwhile, isolates from swine (7/36 [19.44%]), other animals (17/57 [29.82%]), and the natural environment (29/114 [23.68%]) were less prevalent in the cgMLST_200_ groups of human isolates. These results indicate that poultry was the main source of C. coli infection in humans.

### AMR phenotypes in C. coli can effectively be predicted from their genotypes.

To determine whether the antimicrobial susceptibility of C. coli could be predicted using WGS, we analyzed the MICs of C. coli and the presence of antimicrobial resistance determinants (Table 1). Among 141 isolates, 5 were resistant to all 11 antimicrobial agents, and more than half of the isolates were resistant to 8 or more antimicrobial agents (55.32%) (Fig. S1). For MDR, 114 C. coli isolates (80.85%) were resistant to 3 or more classes of antimicrobial agents. Among these, 28 isolates (19.86%) were resistant to all antimicrobial agents. The most prevalent pattern of MDR (48/141 [34.04%]) was resistance to macrolides, fluoroquinolones, aminoglycosides, tetracycline, and lincosamides.

**TABLE 1:** Antimicrobial resistance and distribution of MICs among C. coli isolates

Antimicrobial	No. (%) of resistant isolates (*n* = 141)	No. of isolates with MICs (μg/mL)^*a*^												
		<0.25	0.25	<0.5	0.5	1	2	4	8	16	32	>32	64	>64
Erythromycin	83 (58.87)			6		18	23	9	2	1	3		15	64
Azithromycin	85 (60.28)			56			1	2	1	4	9		13	55
Nalidixic acid	138 (97.87)					1	2						10	128
Ciprofloxacin	138 (97.87)			3		1		2	9	31	53		27	15
Gentamicin	80 (56.74)			19		28	14	1			4		9	66
Streptomycin	99 (70.21)			1		6	13	10	12	6	25		32	36
Chloramphenicol	12 (8.51)			1		4	15	45	48	16	7		3	2
Florfenicol	30 (21.28)			1		14	43	53	20	3	3		1	3
Tetracycline	137 (97.16)			2		2			2	1	2		15	117
Telithromycin	92 (65.25)	2			5	5	18	19	10	2	3	77		
Clindamycin	85 (60.28)	21			25	10	3	9	11	9	9	44		

a Shading indicates antimicrobial resistance.

10.1128/mbio.02835-22.1 FIG S1 Patterns of resistance of 141 isolates of C. coli to various antimicrobial combinations. The *x* axis represents the number (percent) of C. coli isolates. The *y* axis represents a series of combination of antimicrobials. One hundred fourteen (80.85%) C. coli isolates were multidrug resistant. Antimicrobials included erythromycin (ERY), azithromycin (AZI), nalidixic acid (NAL), ciprofloxacin (CIP), gentamicin (GEN), streptomycin (STR), florfenicol (FLO), tetracycline (TET), telithromycin (TEL), and clindamycin (CLI). Download FIG S1, PDF file, 0.8 MB. Copyright © 2022 Zhang et al. 2022 Zhang et al. https://creativecommons.org/licenses/by/4.0/ This content is distributed under the terms of the Creative Commons Attribution 4.0 International license.

**FIG 3:**
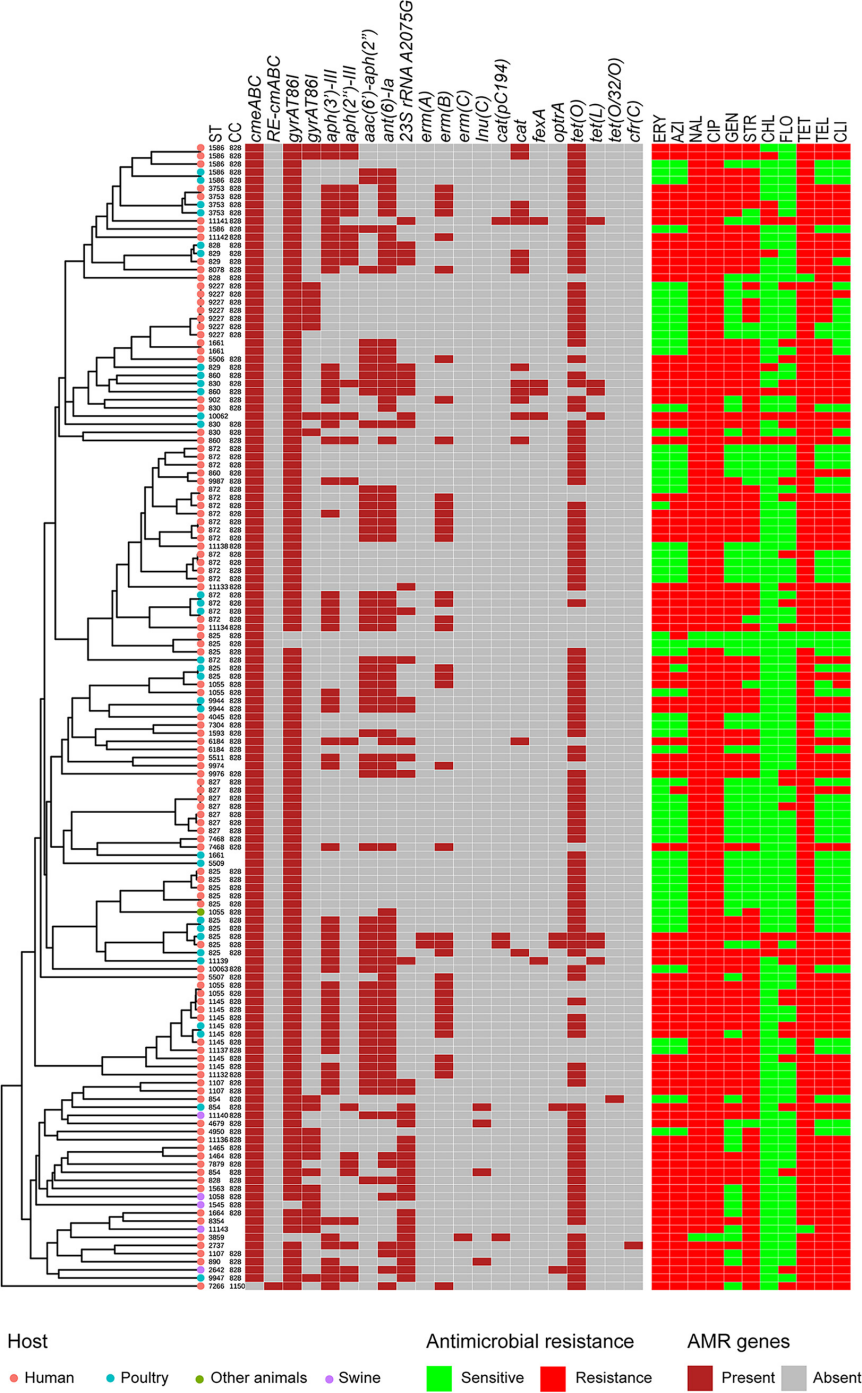
cgMLST showing the lineage, ST, clonal complex, and antimicrobial resistance phenotypes and genotypes of 141 resistant C. coli. ST, MLST type; CC, clonal complex; ERY, erythromycin; AZI, azithromycin; NAL, nalidixic acid; CIP, ciprofloxacin; GEN, gentamicin; STR, streptomycin; FLO, florfenicol; TET, tetracycline; TEL, telithromycin; CLI, clindamycin.

The AMR determinants associated with the six classes of antimicrobial agents were analyzed using ResFinder v. 3.0 (Fig. 3). The overall correlation rate between AMR phenotype and genotype was 92.60% (84.40% to 99.29%), with a sensitivity of 93.03%, specificity of 91.19%, positive predictive value (PPV) of 97.25%, and negative predictive value (NPV) of 79.62% (Table 2). We identified 73 (7.40%) discrepancies, most of which were associated with an absence of resistance determinants among phenotypically resistant isolates, particularly for those resistant to tetracycline and streptomycin. The highest correlation was found for ciprofloxacin and nalidixic acid resistances, which are associated with the *gyrA* T86I point mutation. Isolates containing either the *erm*(B) gene or the A2075G mutation in 23S rRNA were considered macrolide resistant. The rates of correlation of erythromycin and azithromycin resistance to the *erm*(B) gene and the A2075G mutation were 96.45% and 95.04%, respectively (Table 2). A previous study showed a 98.8% overall correlation rate between genotype resistance based on WGS and phenotypic resistance in C. coli, proving that genotypic resistance can be used to predict phenotype with high accuracy (20).

**TABLE 2:** Comparison between agar dilution-based phenotypic resistance and antimicrobial resistance mechanism according to AMR genetic determinants for 141 C. coli isolates

Antimicrobial	Phenotypic profile	No. (%) of isolates	AMR determinant(s) detected	No. with AMR gene present/absent	Correlation rate^*a*^ (%)	Sensitivity^*b*^ (%)	Specificity^*c*^ (%)	PPV^*d*^ (%)	NPV^*e*^ (%)
Ciprofloxacin and nalidixic acid	Resistant	138 (97.87)	*gyrA* T86I	137/1	99.29	99.28	100	100	75.00
	Susceptible	3 (2.13)		0/3					
Gentamicin	Resistant	80 (56.74)	*aph(3′)-III* and/or *aac(6′)-aph(2′')* and/or *ant(6)-Ia*	77/3	89.36	96.25	80.33	86.52	94.23
	Susceptible	61 (43.26)		12/49					
Streptomycin	Resistant	99 (70.21)	*aph(3′)-III* and/or *aac(6′)-aph(2′')* and/or *ant(6)-Ia*	83/16	84.40	83.84	85.71	93.26	69.23
	Susceptible	42 (29.79)		6/36					
Tetracycline	Resistant	137 (97.16)	*tet*(O)	115/22	84.40	83.94	100	100	16.67
	Susceptible	4 (2.84)		0/4					
Erythromycin	Resistant	83 (58.87)	23S rRNA A2075G and/or *erm*(B)	79/4	96.45	95.18	98.28	98.75	93.44
	Susceptible	58 (42.13)		1/57					
Azithromycin	Resistant	85 (60.28)	23S rRNA A2075G and/or *erm*(B)	79/6	95.04	92.94	98.21	98.75	90.16
	Susceptible	56 (39.72)		1/55					
Total^*f*^	Resistant	760 (77.00)		707/53	92.60	93.03	91.19	97.25	79.62
	Susceptible	227 (23.00)		20/207					

a Calculated as the sum of true positives and true negatives divided by all tested isolates.

b Calculated by dividing true positives by the sum of true positives and false positives.

c Calculated by dividing true negatives by the sum of true negatives and false negatives.

d Calculated by dividing true positives by the sum of true positives and false negatives.

e Calculated by dividing true negatives by the sum of true negatives and false positives.

f For gentamicin and streptomycin, *aph(3′)-III* and/or *aac(6′)-aph(2′')* and/or *ant(6)-Ia* was used for calculation. For erythromycin and azithromycin, 23S rRNA A2075G and/or *erm*(B) was used for calculation.

### Increased resistance to multiple antimicrobial agents among C. coli isolates, as predicted by WGS.

**FIG 4:**
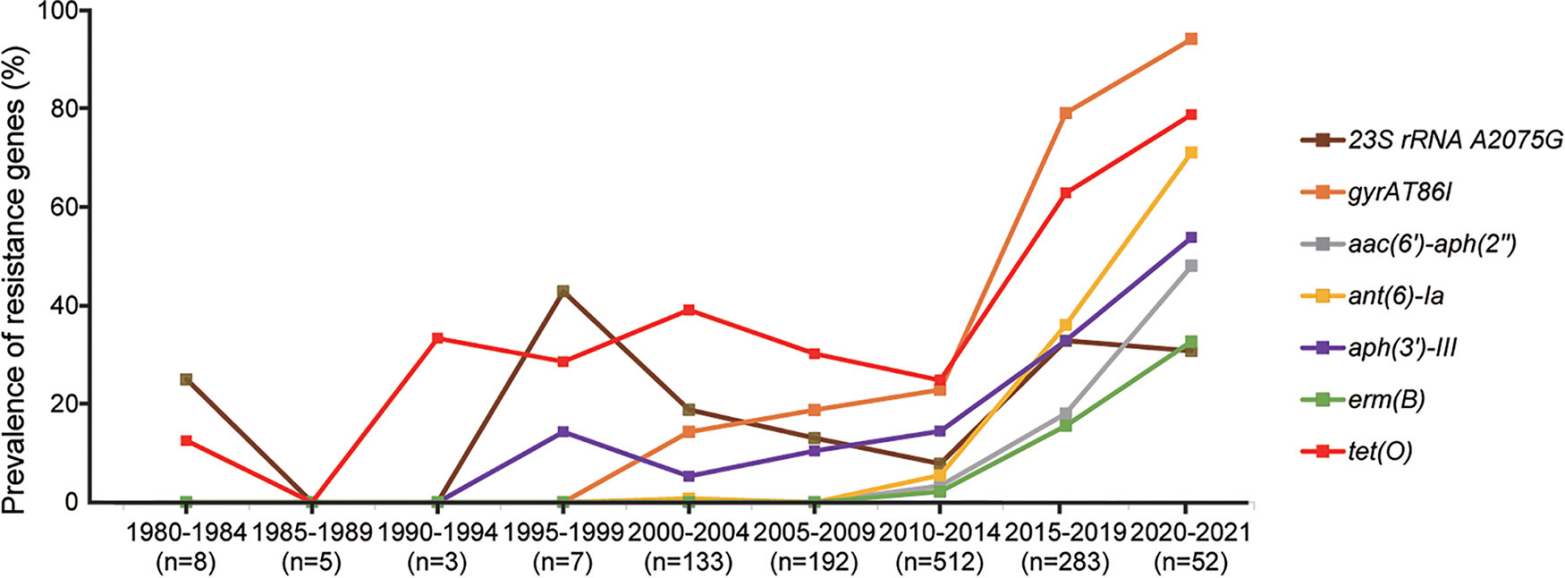
Trends in the prevalence of *tet*(O), *aph(3′)-III*, *aac(6′)-aph(2′')*, *ant(6)-Ia*, *gyrA* T86I, *erm*(B), and 23S rRNA A2075G in C. coli isolates from GenBank and our lab.

To characterize temporal changes in AMR, we analyzed the presence of AMR determinants in C. coli sequences collected since the 1980s in public databases (*n* = 1,195). These included 38 acquired AMR genes and 3 resistance-conferring point mutations (Table 3). Over the past 40 years, the prevalence of AMR mechanisms has increased significantly (Fig. 4). The prevalence of the 23S rRNA A2075G mutation and *erm*(B) gene was 30.77% and 32.69% in 2020 to 2021, respectively. The prevalence of *aph*(*3′*)*-III*, *aac*(*6′*)*-aph*(*2″*), and *ant(6*)*-Ia* increased from 0.00% in 1980 to 1990 to 53.85%, 48.08%, and 71.15% in 2020 to 2021, respectively. The prevalence of *tet*(O) increased 10-fold from 1980–1990 to 2020–2021 (78.85%). The *gyrA* T86I point mutation had the highest prevalence rate in 2020 to 2021 (94.23%) (Fig. 4).

**TABLE 3:** Antimicrobial resistance mechanisms detected in 1,195 C. coli isolates^*a*^

Antimicrobial class	Genetic AMR determinant	Description	No. (%) of positive isolates
Tetracyclines	*tet*(O)	Acquired AMR gene	460 (38.49)
	*tet*(L)	Acquired AMR gene	20 (1.67)
	*tet*(O/32/O)	Acquired AMR gene	19 (1.59)
	*tet*(W)	Acquired AMR gene	12 (1.00)
	*tet*(O/W/32/O)	Acquired AMR gene	1 (0.08)
Fluoroquinolones	*gyrA* T86I	Point mutation	445 (37.24)
Aminoglycoside	*aph(3′)-III*	Acquired AMR gene	223 (18.66)
	*ant(6)-Ia*	Acquired AMR gene	168 (14.06)
	*aadE-Cc*	Acquired AMR gene	161 (13.47)
	*aac(6′)-aph(2′')*	Acquired AMR gene	94 (7.87)
	*aph(2′')-If*	Acquired AMR gene	49 (4.10)
	*aph(2′')-Ig*	Acquired AMR gene	39 (3.26)
	*aph(2′')-Ic*	Acquired AMR gene	14 (1.17)
	*aph(3′)-VIIa*	Acquired AMR gene	11 (0.92)
	*aph(2′')-Ib*	Acquired AMR gene	5 (0.42)
	*aac(6′)-Im*	Acquired AMR gene	5 (0.42)
β-Lactams	*bla*_OXA-489_	Acquired AMR gene	676 (56.57)
	*bla*_OXA-193_	Acquired AMR gene	407 (34.06)
	*bla*_OXA-450_	Acquired AMR gene	387 (32.38)
	*bla*_OXA-453_	Acquired AMR gene	387 (32.38)
	*bla*_OXA-61_	Acquired AMR gene	382 (31.97)
	*bla*_OXA-452_	Acquired AMR gene	378 (31.63)
	*bla*_OXA-451_	Acquired AMR gene	371 (31.05)
	*bla*_OXA-460_	Acquired AMR gene	84 (7.03)
	*bla*_OXA-461_	Acquired AMR gene	42 (3.51)
	*bla*_OXA-465_	Acquired AMR gene	1 (0.08)
	*bla*_TEM-116_	Acquired AMR gene	1 (0.08)
	*bla*_TEM-229_	Acquired AMR gene	1 (0.08)
Macrolides	23S rRNA A2075G	Point mutation	204 (17.07)
	23S rRNA A2074G	Point mutation	2 (0.17)
	*erm*(A)	Acquired AMR gene	8 (0.67)
	*erm*(B)	Acquired AMR gene	72 (6.03)
Lincosamides	*lnu*(B)	Acquired AMR gene	1 (0.08)
	*lnu*(C)	Acquired AMR gene	14 (1.17)
	*lsa*(E)	Acquired AMR gene	1 (0.08)
Phenicols	*cat*	Acquired AMR gene	30 (2.51)
	*cat*(pC194)	Acquired AMR gene	12 (1.00)
	*optrA*	Acquired AMR gene	12 (1.00)
	*fexA*	Acquired AMR gene	7 (0.59)
Multidrug efflux transporter	*cmeABC*	Acquired AMR gene	1181 (98.83)
	*RE-cmeABC*	Acquired AMR gene	11 (0.92)

a Mutation reference isolates were *gyrA*-like ATCC 33559 and 23S rRNA-like NCTC 11168.

**FIG 5:**
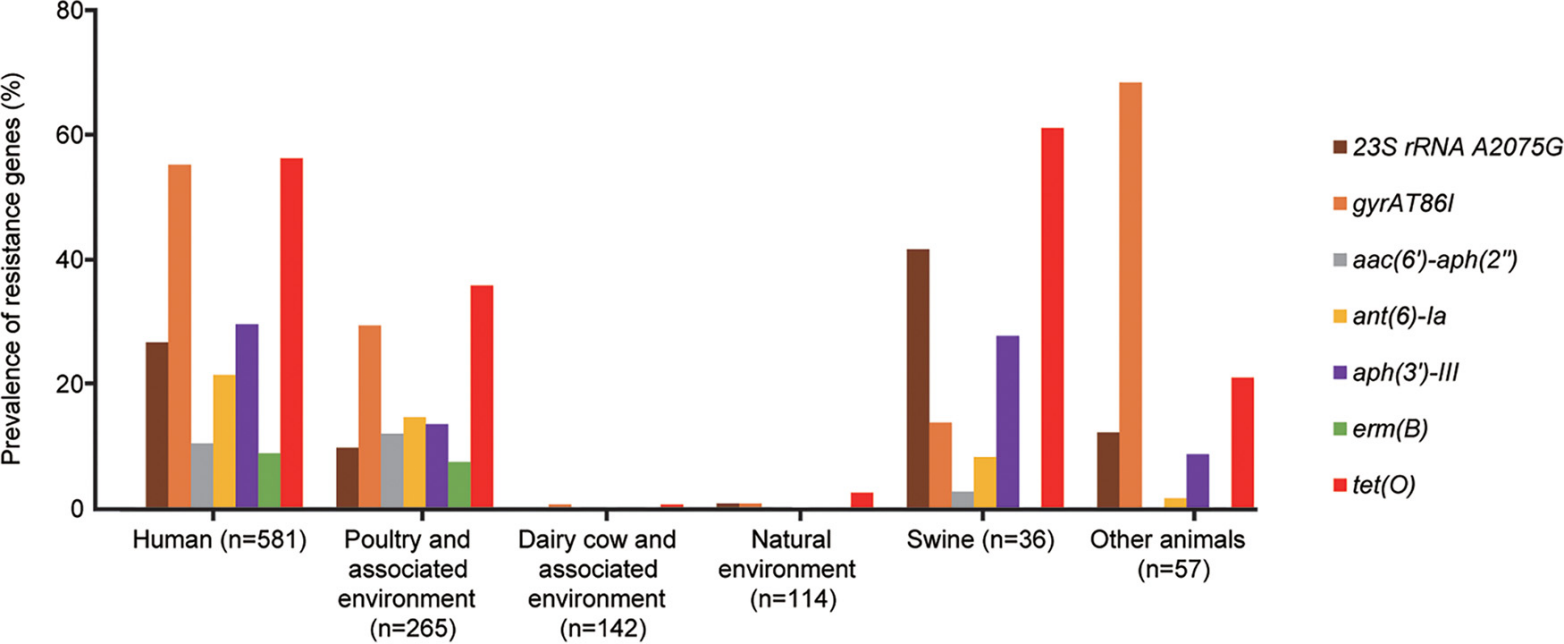
Prevalence of antimicrobial resistance mechanisms in C. coli isolates compared by source.

In general, isolates from humans as well as poultry and the associated environment showed a significant increase in AMR mechanisms (Fig. S2 and S3). The AMR profiles of human and poultry isolates were similar (Fig. 5). The *erm*(B) gene was found only in isolates from humans as well as poultry and the associated environment. There were few resistance mechanisms in dairy cattle and natural-environment isolates, and only 2.63% of samples from natural environments possessed *tet*(O) (Fig. 5). *tet*(O) was most common among isolates from humans, swine, and poultry and the associated environment (56.28%, 61.11%, and 35.85%, respectively). We compared the detection rates of AMR mechanisms between humans and poultry and the associated environment from 2015 to 2021 and found that *gyrA* T86I, *erm*(B), and three aminoglycoside resistance genes were more common in poultry than in human C. coli isolates (Fig. S4).

10.1128/mbio.02835-22.2 FIG S2 Prevalence of *tet*(O), *aph(3′)-III*, *aac(6′)-aph(2″)*, *ant(6)-Ia*, *gyrA* T86I, *erm*(B), and 23S rRNA A2075G in C. coli isolates from humans. Download FIG S2, PDF file, 0.9 MB. Copyright © 2022 Zhang et al. 2022 Zhang et al. https://creativecommons.org/licenses/by/4.0/ This content is distributed under the terms of the Creative Commons Attribution 4.0 International license.

10.1128/mbio.02835-22.3 FIG S3 Prevalence of *tet*(O), *aph(3′)-III*, *aac(6′)-aph(2″)*, *ant(6)-Ia*, *gyrA* T86I, *erm*(B), and 23S rRNA A2075G in C. coli isolates from poultry and the associated environment. Download FIG S3, PDF file, 0.9 MB. Copyright © 2022 Zhang et al. 2022 Zhang et al. https://creativecommons.org/licenses/by/4.0/ This content is distributed under the terms of the Creative Commons Attribution 4.0 International license.

10.1128/mbio.02835-22.4 FIG S4 Comparison of antimicrobial resistance mechanisms between C. coli isolates from human and from poultry and the associated environment in 2015 to 2021 (a) and common STs (b to g). NA, *P* ≥ 0.05; *, *P* < 0.05; **, *P* < 0.01; ***, *P* < 0.001. The numbers of isolates between humans and poultry and the associated environment were 260 and 44 (a), 39 and 31 (b), 27 and 27 (c), 10 and 16 (d), 52 and 6 (e), 47 and 6 (f), and 3 and 28 (g). Download FIG S4, PDF file, 1.0 MB. Copyright © 2022 Zhang et al. 2022 Zhang et al. https://creativecommons.org/licenses/by/4.0/ This content is distributed under the terms of the Creative Commons Attribution 4.0 International license.

The prevalence of antimicrobial resistance mechanisms in C. coli from China was higher than that from other countries. The prevalence of AMR mechanisms other than 23S rRNA A2075G was higher in China than in other countries (*n* = 1,192 [three isolates with no clear source location were removed]) (Fig. S5). From 2014 to 2018, the detection rate of AMR mechanisms other than *erm*(B) (0% in the United States before 2018) and 23S rRNA A2075G was higher in China than in the United States (Table 4). Furthermore, the prevalence of *erm*(B) in human isolates from China increased from 26.04% (26/96) in 2015 to 2019 to 55.00% (11/20) in 2020 to 2021. The *cmeABC* variant *RE-cmeABC* was found in 11 isolates. Ten of the 11 human isolates were from Lima, Peru, between 2010 and 2017.

**TABLE 4:** Comparison of antimicrobial resistance mechanisms between China and U.S. human C. coli isolates from 2014 to 2018

AMR determinant detected	No. (%) of human cases		χ^2^	*P*
	China (*n* = 43)	USA (*n* = 19)		
23S rRNA A2075G	11 (25.58)	9 (47.37)	2.8624	0.1399
*gyrA* T86I	40 (93.02)	7 (36.84)	22.6781	<0.0001
*aac(6′)-aph(2′')*	13 (30.23)	0 (0.00)	7.2682	0.0045
*ant(6)-Ia*	18 (41.86)	1 (5.26)	8.3041	0.0059
*aph(3′)-III*	13 (30.23)	1 (5.26)	4.6996	0.0459
*erm*(B)	8 (18.60)	0 (0.00)	4.0586	0.0932
*tet*(O)	34 (79.07)	4 (21.05)	18.6953	<0.0001

10.1128/mbio.02835-22.5 FIG S5 The prevalence of *tet*(O), *aph(3′)-III*, *aac(6′)-aph(2″)*, *ant(6)-Ia*, *gyrA T86I*, *erm*(B), and 23S rRNA A2075G in C. coli isolates in different countries. Other regions include only three isolates for 2020 to 2021, and the United Kingdom had only two isolates for 2015 to 2019. Download FIG S5, PDF file, 1.0 MB. Copyright © 2022 Zhang et al. 2022 Zhang et al. https://creativecommons.org/licenses/by/4.0/ This content is distributed under the terms of the Creative Commons Attribution 4.0 International license.

## DISCUSSION

Our findings suggest that increased resistance in C. coli contributed to the increase in campylobacteriosis caused by C. coli. First, phylogenetic analysis showed that poultry was an important source of human C. coli isolates worldwide. Second, antimicrobials, especially macrolides, florfenicol, fluoroquinolones, and tetracyclines, are widely used in poultry production, promoting the occurrence of antimicrobial-resistant bacteria in China. Third, C. coli has replaced C. jejuni as the dominant Campylobacter species in Chinese poultry products (15). Finally, C. coli was less prevalent in countries where C. coli has lower AMR rates, both in poultry and human isolates (17, 21–24). The use of antimicrobials in poultry production was the most likely cause of the shift in C. coli prevalence. We propose that antimicrobial-resistant C. coli is better adapted than C. jejuni to the poultry industry in China, and increased AMR rates in C. coli resulted in the increase of campylobacteriosis caused by C. coli.

C. jejuni and C. coli account for about 95% and 5%, respectively, of campylobacteriosis cases worldwide. A study of patients with diarrhea in Israel from 1982 to 1985 found that 7.9% of campylobacteriosis was caused by C. coli (25). In Denmark, 6% of Campylobacter isolates from campylobacteriosis patients in 1995 to 1996 were C. coli (26). A study in the United Kingdom in 2000 found that 7% of human campylobacteriosis was caused by C. coli (27) and that the proportion of C. coli detected in children with diarrhea was higher than that in adults. In Poland during 2003 to 2005, the proportion of C. coli detected in children with campylobacteriosis who exhibited diarrhea was 13.6% (28). Ethiopia and Tanzania separately compared adults and children with diarrhea caused by campylobacteriosis and found that the detection rate of C. coli in adults was 3.4% to 5.9%, while that in children was 9.1% to 21.1% (16, 29–32). These findings imply that C. coli accounts for a high proportion of infections in people with lower immune function, such as infants and children. Most recent studies have not shown an increase in the proportion of C. coli. For example, studies in Latvia, Egypt, and Iran showed that the proportion of C. coli did not exceed 7.5% from 2015 to 2018 (18, 21, 33). Therefore, the increasing proportion of C. coli infection among Chinese adults warrants continued consideration (19).

Previous studies have demonstrated that AMR genotypes can be used to predict the resistance phenotype (20). Similarly, we found that the *aph*(*3′*)*-III*, *aac*(*6′*)*-aph*(*2″*), and *ant(6*)*-Ia* genes jointly led to C. coli resistance to aminoglycosides, and the *aadE-Cc* gene also played a major role. The correlation between the *ant(6*)*-Ia* gene and resistance to gentamicin reached 87.23% (data not shown). However, without the synergistic effects of several genes, the correlation of each gene with resistance to streptomycin was very low. When several genetic determinants were considered, the prevalence rates of the *erm*(B) gene and 23S rRNA A2075G mutation were 27.66% and 29.08%, respectively. Both factors jointly mediated resistance to erythromycin (58.87%) and azithromycin (60.28%). The development of this resistance mechanism in C. coli was concerning, because no isolate had both the *erm*(B) gene and the 23S rRNA A2075G mutation.

Antimicrobials are widely used in the production of livestock in China, which has directly led to an increase in the antimicrobial resistance of C. coli (13–15). Some antimicrobial resistance genes have been recurrently identified in C. coli in recent years, including *erm*(B), which is associated with macrolide resistance (34), *fexA* and *optrA*, which are associated with phenicol resistance, *cfr*(C), which is associated with MDR, and a variant of the potent multidrug efflux pump gene operon *cmeABC* (*RE-cmeABC*) (35–38). To date, at least 12 types of multidrug resistance gene islands (MDRGI) have been identified in C. coli (39, 40). Antimicrobial resistance genes have made a significantly higher contribution to antimicrobial resistance of C. coli in China than in other countries (41). We found that C. coli isolates from human and poultry sources were closely related, consistent with findings in China, Germany, Scotland, and France (42–45), indicating that poultry remains the main source of human infection with C. coli. In addition, although the prevalence of AMR mechanisms of C. coli from poultry was higher than that from human, we found similarities in AMR patterns among common STs in human and poultry isolates, providing evidence for zoonotic transmission of AMR between poultry and human (Fig. S4). C. coli is still a dominant Campylobacter species isolated from swine, and the potential contribution of swine C. coli to the increasing human C. coli infections in China requires attention. Previous studies indicated the high prevalence of C. coli in swine farms and associated environment (46, 47). However, we found that the prevalence of C. coli from swine meat was much lower that of isolates from poultry meat (Table S1). In addition, STs of swine isolates were different from those of human isolates. Thus, we believe that swine C. coli is unlikely to contribute to the increased occurrence of campylobacteriosis caused by C. coli in China. In addition, compared with those identified in human and animal C. coli, only a few AMR mechanisms were detected in the natural environment. This emphasizes the importance of the use of antimicrobials in animal breeding and also suggests that the natural environment may not be an important source of human infection with C. coli.

We found that the prevalence of *tet*(O), *aph*(*3′*)*-III*, *aac*(*6′*)*-aph*(*2″*), *ant(6*)*-Ia*, *gyrA* T86I, *erm*(B), and 23S rRNA A2075G in the GenBank sequences of C. coli increased to various degrees in the past 10 years. This is consistent with increasing rates of C. coli resistance to fluoroquinolones, macrolides, tetracyclines, and aminoglycosides (14, 48–50). In addition, we found that the prevalence of resistance mechanisms in C. coli isolates was higher in China than in other countries, which is consistent with the higher rates of C. coli resistance to various antimicrobials in China compared to other countries (47). Therefore, AMR in C. coli is an increasingly serious threat that requires coordinated monitoring and management to minimize the emergence and spread of C. coli with AMR through the food chain.

We acknowledge some limitations to our study design. More than half of the isolates analyzed were from human sources. In terms of geographic representation, most of the isolates were from the United Kingdom, the United States, and China, and the collection dates were unevenly distributed. Nevertheless, our sample sizes were sufficient for determining AMR trends. In future studies, we will consider the fitness cost and compensatory mechanisms related to the increasing proportion of C. coli infection rates in China.

### Conclusions.

The continued use of macrolides, florfenicol, fluoroquinolones, and tetracyclines in the Chinese poultry industry promotes the survival of antimicrobial-resistant bacteria. Preliminary studies have shown that antimicrobial-resistant C. coli replaced C. jejuni as the dominant Campylobacter species in poultry products. In this study, we found that human C. coli isolates originated mainly from poultry. Therefore, we propose that the degree of AMR determines the proportion of C. coli infections in humans. Given that the AMR level of C. coli is much higher than that of C. jejuni, the treatment of campylobacteriosis in humans that are infected with highly resistant C. coli is expected to become more difficult. Therefore, we recommend using antimicrobials rationally in poultry breeding and production, as reducing the selective fitness of C. coli will reduce the risk of infection in humans.

## MATERIALS AND METHODS

### Ethical considerations.

The study protocol was approved by the ethics committee of the Beijing Center for Disease Control and Prevention. Participants received information about the purpose of the study and their right to confidentiality. Written informed consent was obtained from each participant or the participant’s parent or guardian (in the case of children).

### Sample collection and bacterial isolation.

From 2016 to 2021, Campylobacter isolates were obtained from patients with acute diarrhea in 19 hospitals in Beijing. Patients were defined as having three or more instances of watery, loose, mucosal, or bloody stools over a 24-h period. Five milligrams of fresh stool sample was collected from each person with diarrhea. Animal meat was collected in eight retail outlets and 12 supermarkets in Beijing in 2018 and 2021; the samples consisted of mainly fresh poultry (including broilers and ducks), supplemented with samples from swine and other animals. All samples were sent to the laboratory for bacterial isolation within 24 h.

Two Campylobacter isolation kits based on the membrane filter method (ZC-CAMPY-001 and ZC-CAMPY-002; Qingdao Sinova Biotechnology Co., Ltd., Qingdao, China) were used to isolate Campylobacter from meat samples and human stool samples, respectively. Briefly, meat samples were combined with buffered peptone water and agitated for 5 min; 2 mL buffered peptone water or 1 mL of fecal specimen suspension was then transferred to 4 mL of the enrichment medium provided in the kit. The main component of the enrichment medium was a modified Preston broth. The enrichment medium was then cultured for 48 h at 42°C in a microaerobic atmosphere (5% O_2_, 10% CO_2_, and 85% N_2_). Approximately 300 μL of enrichment medium was spotted on the membrane filter (0.45 μm) surface of the kit and spread onto Columbia and Karmali agar plates. Five or more colonies (or all colonies if fewer than five were present) resembling Campylobacter were picked after 48 h of incubation at 42°C in a microaerobic atmosphere. All isolates were first identified with a Vitek 2 compact system (bioMérieux, Lyons, France) or by matrix-assisted laser desorption ionization–time of flight mass spectrometry (Bruker, Leipzig, Germany) and further verified by PCR, according to a previously described method (51).

### Antimicrobial susceptibility testing.

The MICs of C. coli isolates were determined according to an agar dilution method, as recommended by the Clinical and Laboratory Standards Institute, using a commercial kit (Zhongchuang Biotechnology, Qingdao, China). Six classes of antimicrobial agents were chosen for this study: macrolides, fluoroquinolones, aminoglycosides, chloramphenicols, tetracyclines, and lincosamides. C. jejuni ATCC 33560 was used as the reference isolate. The cutoffs for resistance used in this study were based on standards from the National Antimicrobial Resistance Monitoring System (last visited 28 August 2021), and MDR was defined as resistance to three or more classes of antimicrobial agents.

### Genomic DNA extraction and whole-genome sequencing.

Genomic DNA was extracted with a commercial kit (QIAamp DNA minikit; Qiagen GmbH, Hilden, Germany) according to the manufacturer’s protocol. Sequencing was performed with an Illumina NovaSeq apparatus (Illumina, San Diego, CA, USA) and two paired-end libraries with average insertion lengths of 350 bp and 2,000 bp. Raw data were processed in several steps by removing reads with 5 bp of ambiguous bases, reads with 20 bp of low-quality (*Q*_20_) bases, adapter contamination, and duplicated reads. Finally, 100× libraries were obtained with clean paired-end read data. Raw WGS data were imported into BioNumerics v. 7.6 (Applied Maths, Oost-Vlaanderen, Belgium) and then uploaded to the National Molecular Tracing Network for Foodborne Diseases Surveillance (TraNet) calculation engine at Aliyun (Alibaba Group, Hangzhou, China) for *de novo* assembly.

### Genomic analysis.

WGS data for C. coli from both our lab and GenBank were analyzed using ResFinder v. 3.0 (52) with an in-house script for resistance gene identification. BioNumerics v. 7.6 (Applied Maths NV, Sint-Martens-Latem, Belgium) with the Sequence Extraction plug-in and BLAST were used to analyze *gyrA* (C257T, encoding Thr-86-Ile) and 23S rRNA (A2075G). For resistance gene identification, we analyzed the presence of genes mediating resistance to tetracyclines, fluoroquinolones, aminoglycosides, β-lactams, macrolides, lincosamides, and phenicols.

A cgMLST spanning tree was created in BioNumerics v. 7.6 by using categorical differences and the unweighted pair group method with arithmetic means. The cgMLST scheme included 1,343 loci and seven MLSTs (*aspA*, *glnA*, *gltA*, *glyA*, *pgm*, *tkt*, and *uncA*) for C. coli (http://pubmlst.org/campylobacter/). Campylobacter isolates with 200 or more allele differences in the core genome were divided into separate groups.

The genetic determinants of resistance identified by WGS were compared with the phenotypic resistance to seven clinically relevant antimicrobial agents (erythromycin, azithromycin, ciprofloxacin, nalidixic acid, tetracycline, gentamicin, and streptomycin) using the following calculations: correlation (calculated as the sum of true positives and true negatives divided by all tested isolates), sensitivity (calculated by dividing the true positives by the sum of true positives and false positives), specificity (calculated by dividing the true negatives by the sum of true negatives and false negative), PPV (calculated by dividing the true positives by the sum of true positives and false negatives), and NPV (calculated by dividing the true negatives by the sum of true negatives and false positives).

### Statistical analysis.

SPSS software v. 20.0 (IBM SPSS, Chicago, IL, USA) was used for statistical analysis. Differences in the frequencies of antimicrobial resistance mechanisms among C. coli isolates were examined using χ^2^ and Fisher’s exact tests for dichotomous variables; *P* values of <0.05 were considered statistically significant.

### Data availability.

The data sets presented in this study are available in online repositories; the names of the repositories and accession number(s) can be found at NCBI BioProject PRJNA779803.
