# Supplementary material for: Genomic Insights into the Increased Occurrence of Campylobacteriosis Caused by Antimicrobial-Resistant Campylobacter coli
Source: mBio. 2022 Dec 6;13(6):e02835-22. doi: 10.1128/mbio.02835-22 (PMC9765411; doi:10.1128/mbio.02835-22)
Supplement: TABLE S1 [file mbio.02835-22-s0006.docx]

| Sample type | Total no. of samples analyzed | No. (%) of samples with: | | |
| --- | --- | --- | --- | --- |
|  |  | Any *Campylobacter* spp. | *C. jejuni* | *C. coli* |
| Human stool | 11125 | 683 (6.14) | 586 (5.27) | 104 (0.93) |
| Poultry | 158 | 55 (34.81) | 29 (18.35) | 31 (19.62) |
| Broilers | 146 | 49 (33.56) | 25 (17.12) | 29 (19.86) |
| Ducks | 12 | 6 (50.00) | 4 (33.33) | 2 (16.67) |
| Swine | 47 | 5 (10.64) | ND | 5 (10.64) |
| Other animals | 26 | 3 (11.54) | 2 (7.69) | 1 (3.85) |
| Total | 11356 | 746 (6.57) | 617 (5.43) | 141 (1.24) |

Supplementary Table S1. Prevalence of *Campylobacter* isolates detected in patients and animal meats in Beijing.

*ND, Not detected
