# Supplementary material for: Genomic Insights into the Increased Occurrence of Campylobacteriosis Caused by Antimicrobial-Resistant Campylobacter coli
Source: mBio. 2022 Dec 6;13(6):e02835-22. doi: 10.1128/mbio.02835-22 (PMC9765411; doi:10.1128/mbio.02835-22)
Supplement: TABLE S2 [file mbio.02835-22-s0007.docx]

Supplementary Table S2. Summary of *C. coli* isolates recovered in this study and downloaded from GenBank

| Sample type | | Year | | | | | |  |
| --- | --- | --- | --- | --- | --- | --- | --- | --- |
| This study | | 2016 | 2017 | 2018 | 2019 | 2020 | 2021 | Total |
| Human stool | | 1 | 7 | 32 | 48 | 15 | 1 | 104 |
| Poultry | |  |  | 10 |  |  | 21 | 31 |
| Swine | |  |  |  |  |  | 5 | 5 |
| Other animals | |  |  |  |  |  | 1 | 1 |
| Total | | 1 | 7 | 42 | 48 | 15 | 28 | 141 |
| Year | | | | | | | | |
| GenBank | 1980-1989 | 1990-1999 | 2000-2004 | 2005-2009 | 2010-2014 | 2015-2019 | 2020-2021 | Total |
| Human | 10 | 6 | 23 | 95 | 187 | 149 | 7 | 477 |
| Poultry and associated environment |  |  | 64 | 25 | 132 | 11 | 2 | 234 |
| Dairy cow and associated environment |  |  |  |  | 142 |  |  | 142 |
| Natural environment |  | 1 | 16 | 63 | 34 |  |  | 114 |
| Swine | 3 | 3 | 12 | 5 | 5 | 3 |  | 31 |
| Other animals |  |  | 18 | 4 | 12 | 22 |  | 56 |
| Total | 13 | 10 | 133 | 192 | 512 | 185 | 9 | 1054 |
