# Supplementary material for: Genomic Insights into the Increased Occurrence of Campylobacteriosis Caused by Antimicrobial-Resistant Campylobacter coli
Source: mBio. 2022 Dec 6;13(6):e02835-22. doi: 10.1128/mbio.02835-22 (PMC9765411; doi:10.1128/mbio.02835-22)
Supplement: TABLE S3 [file mbio.02835-22-s0008.docx]

Supplementary Table S3. Geographic location and source information of *C. coli* and *C. jejuni* in GenBank

| *C. coli*  Geographic location | Isolation source | | | | | | | | Total |
| --- | --- | --- | --- | --- | --- | --- | --- | --- | --- |
|  | Human | Poultry | Poultry associated environment | Dairy cow | Dairy cow associated environment | Natural environment | Swine | Other animals |  |
| Australia | 42 |  |  |  |  |  | 1 |  | 43 |
| Belgium | 1 |  |  |  |  |  | 1 |  | 2 |
| Bosnia and Herzegovina | 1 |  |  |  |  |  |  |  | 1 |
| Canada | 17 | 4 |  |  |  |  |  |  | 21 |
| Chile | 12 |  |  |  |  |  |  |  | 12 |
| China | 31 | 13 |  |  |  |  |  |  | 44 |
| Colombia | 3 |  |  |  |  |  |  |  | 3 |
| Denmark | 1 | 2 |  |  |  |  |  |  | 3 |
| Finland | 3 |  |  |  |  |  |  |  | 3 |
| France | 50 |  |  |  |  |  |  |  | 50 |
| Germany |  | 4 |  |  |  |  |  |  | 4 |
| India | 1 |  |  |  |  |  |  |  | 1 |
| Ireland | 13 | 2 |  |  |  |  |  |  | 15 |
| Israel | 2 |  |  |  |  |  |  |  | 2 |
| Japan | 2 |  |  |  |  |  | 4 |  | 6 |
| Lebanon | 1 |  |  |  |  |  |  |  | 1 |
| Peru | 25 |  |  |  |  |  | 2 | 1 | 28 |
| Poland | 3 |  |  |  |  |  |  |  | 3 |
| Slovenia | 2 |  |  |  |  |  |  |  | 2 |
| South Korea | 1 |  |  |  |  |  |  |  | 1 |
| Spain |  | 1 |  |  |  |  |  |  | 1 |
| Sweden | 9 |  |  |  |  |  |  |  | 9 |
| Switzerland | 4 | 2 |  |  |  |  |  | 9 | 15 |
| United Kingdom | 119 | 177 | 21 | 49 | 93 | 114 | 12 | 13 | 598 |
| USA | 134 | 9 |  |  |  |  | 11 | 32 | 186 |
| Total | 477 | 214 | 21 | 49 | 93 | 114 | 31 | 55 | 1054 |
|  |  |  |  |  |  |  |  |  |  |
|  |  |  |  |  |  |  |  |  |  |
|  |  |  |  |  |  |  |  |  |  |
| *C. jejuni*  Geographic location | Isolation source | | | | | | | | Total |
|  | Human | | | Poultry | | | Poultry associated environment | |  |
| Australia | 6 | | |  | | |  | | 6 |
| Cambodia | 3 | | |  | | |  | | 3 |
| Canada | 7 | | | 5 | | |  | | 12 |
| China | 52 | | | 78 | | |  | | 130 |
| France | 4 | | | 1 | | |  | | 5 |
| Germany | 1 | | |  | | |  | | 1 |
| Ireland | 11 | | | 8 | | |  | | 19 |
| Peru | 23 | | | 4 | | |  | | 27 |
| Sweden | 1 | | |  | | |  | | 1 |
| United Kingdom | 127 | | | 8 | | | 6 | | 141 |
| USA | 140 | | | 13 | | |  | | 153 |
| Total | 375 | | | 117 | | | 6 | | 498 |
