# Supplementary figures and images for: Genomic Insights into the Increased Occurrence of Campylobacteriosis Caused by Antimicrobial-Resistant Campylobacter coli
Source: mBio. 2022 Dec 6;13(6):e02835-22. doi: 10.1128/mbio.02835-22 (PMC9765411; doi:10.1128/mbio.02835-22)

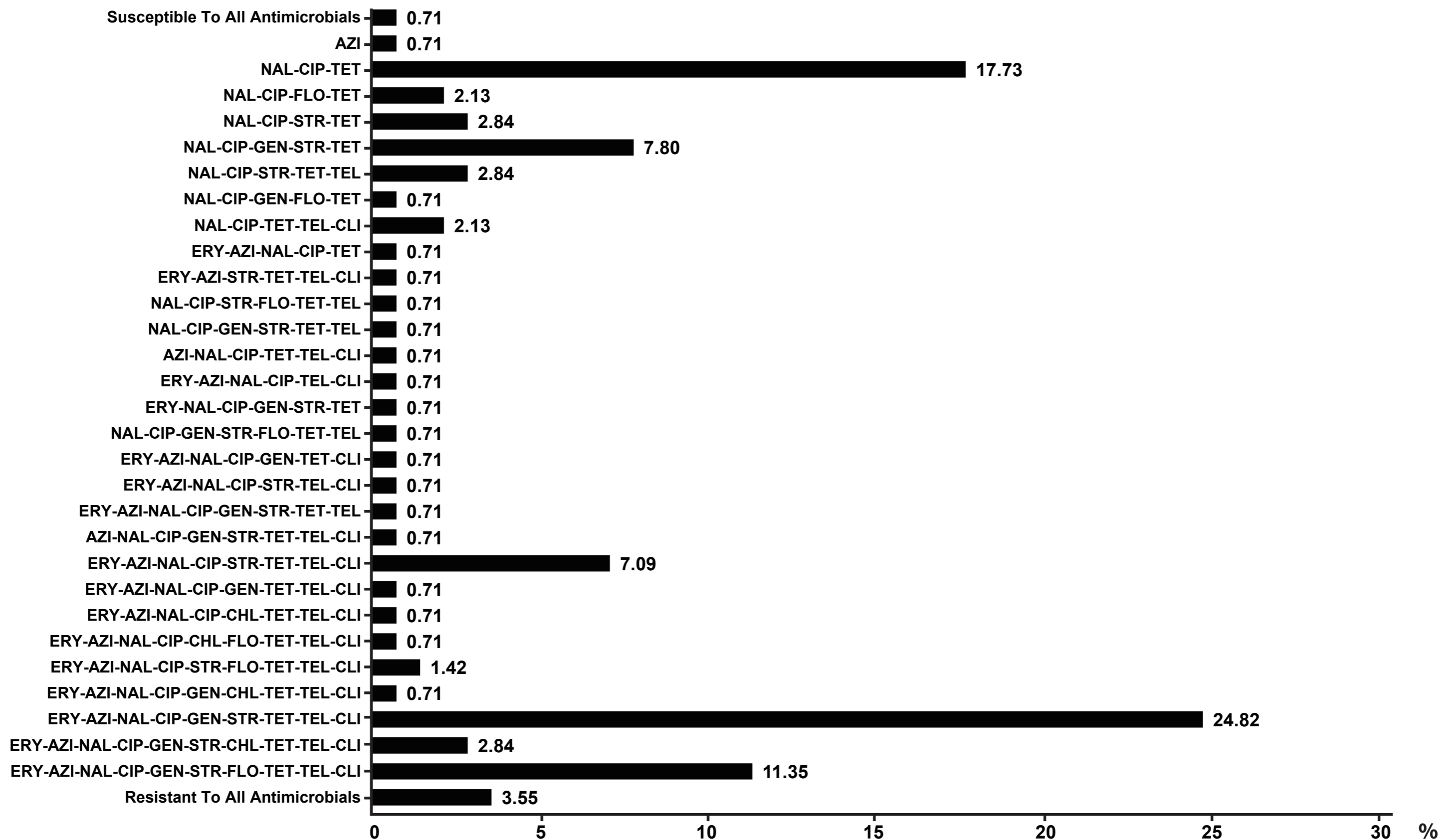

Supplement: FIG S1 [file mbio.02835-22-s0001.pdf]

# Human

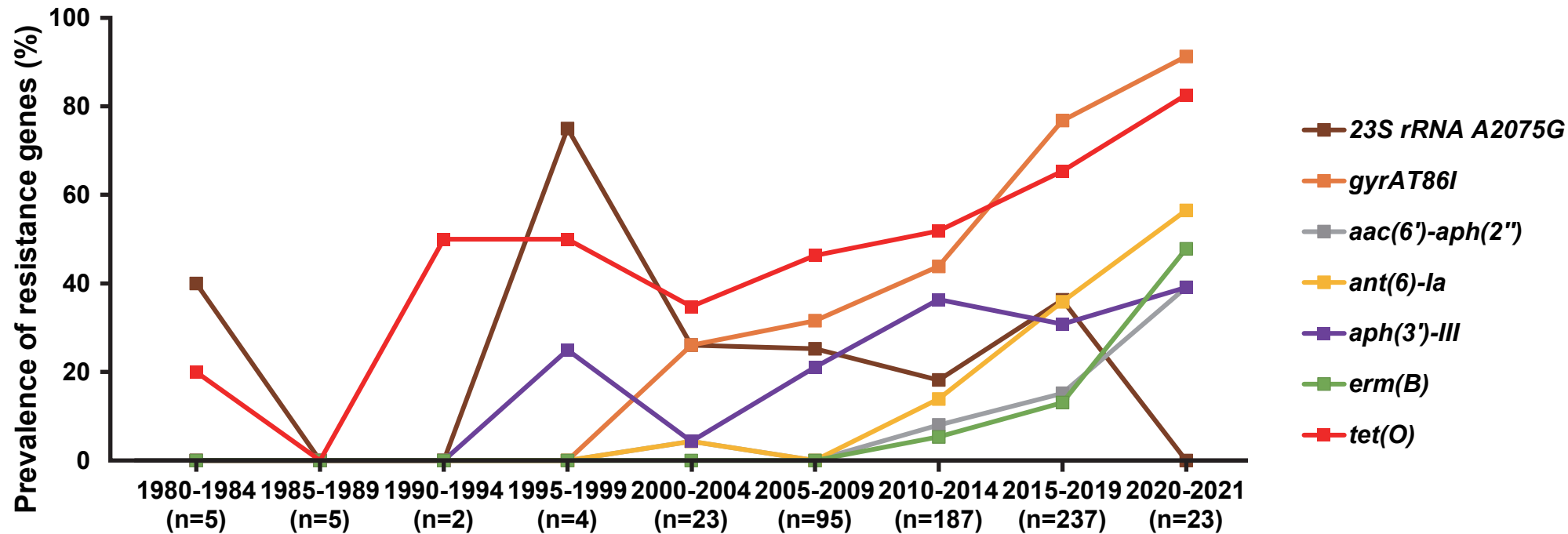

Supplement: FIG S2 [file mbio.02835-22-s0002.pdf]

## Poultry and associated environment

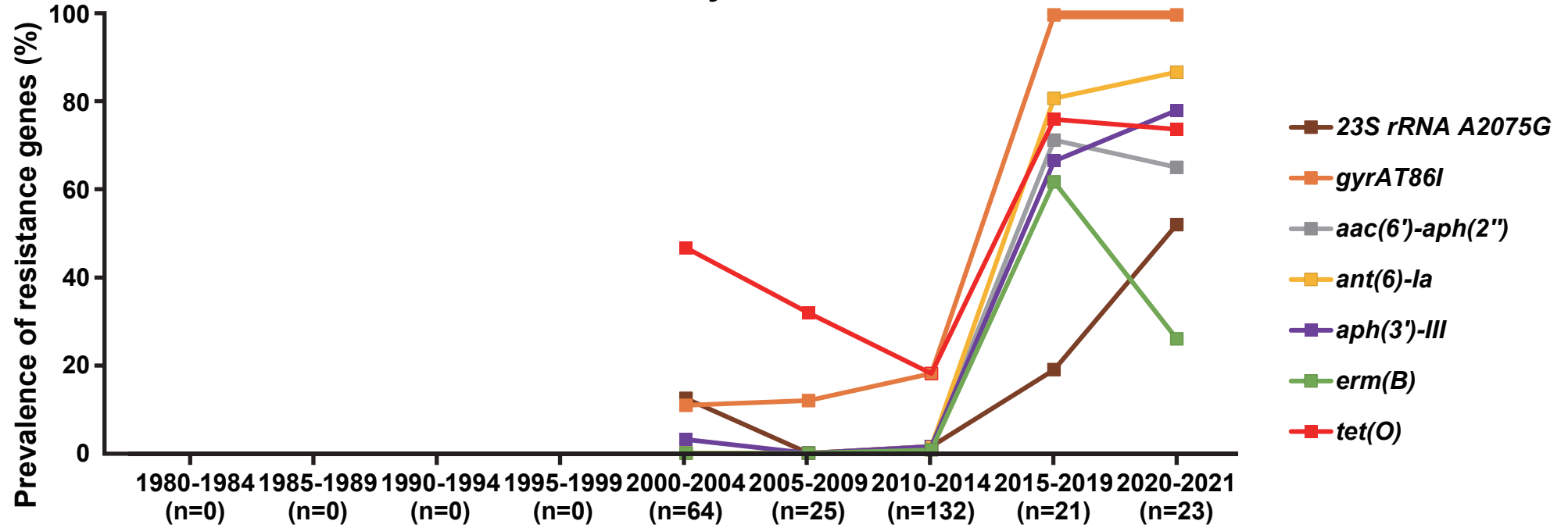

Supplement: FIG S3 [file mbio.02835-22-s0003.pdf]

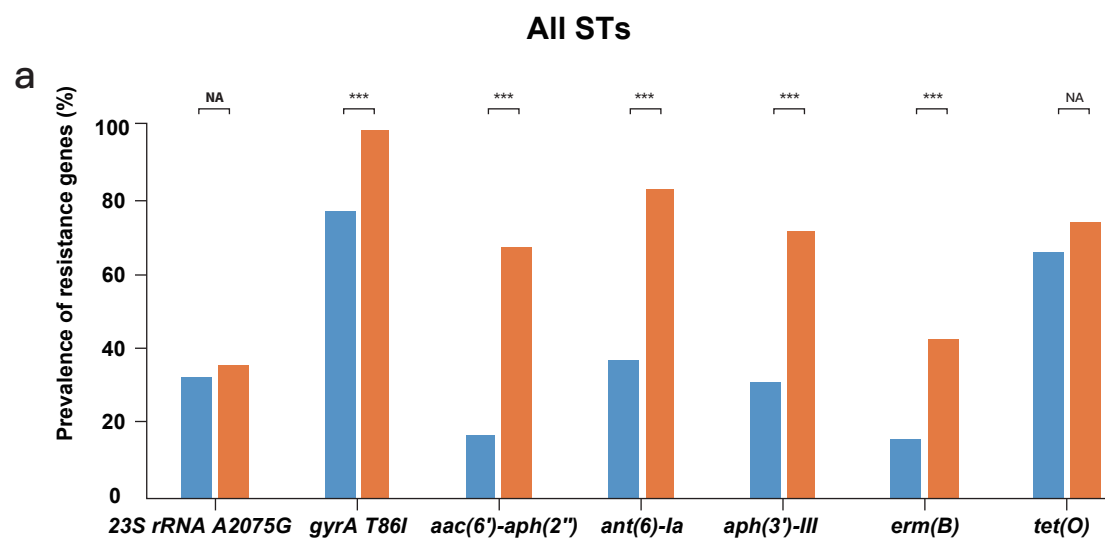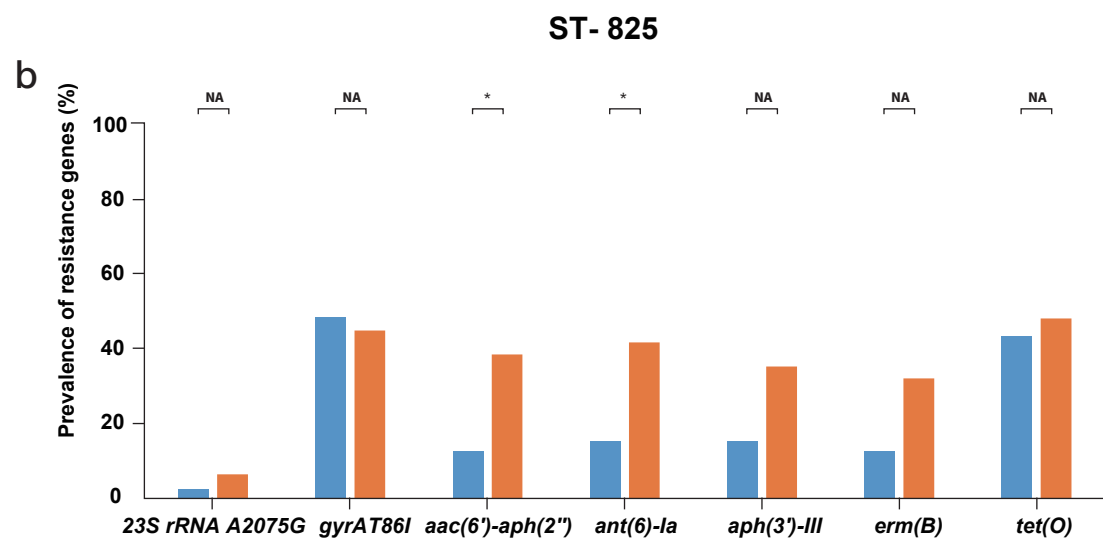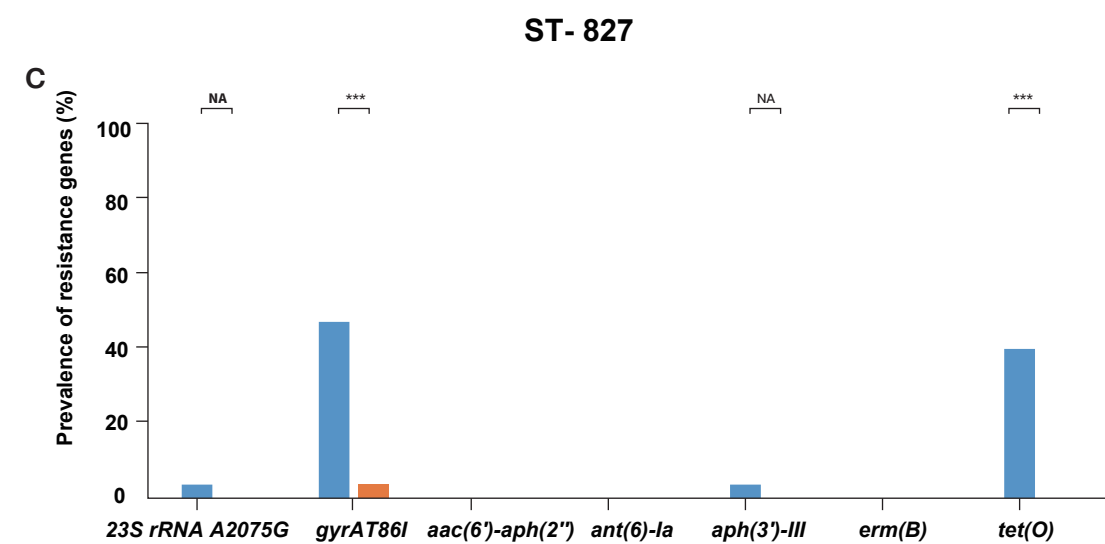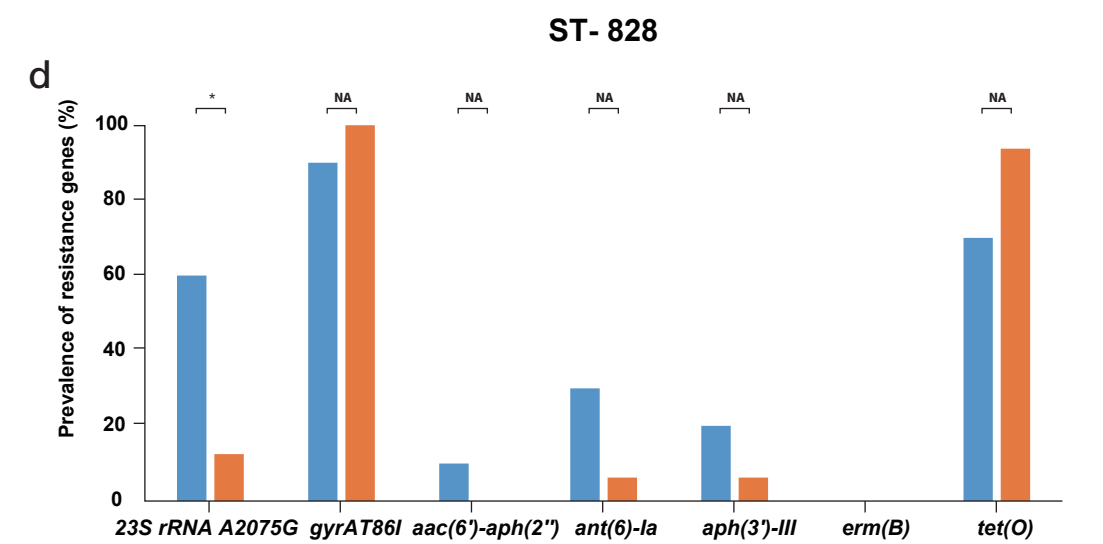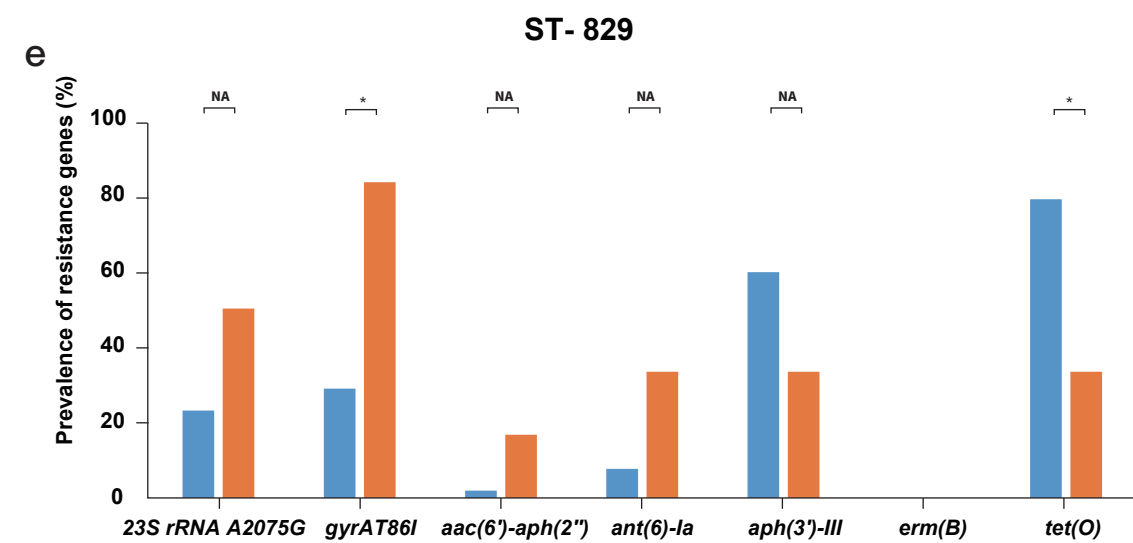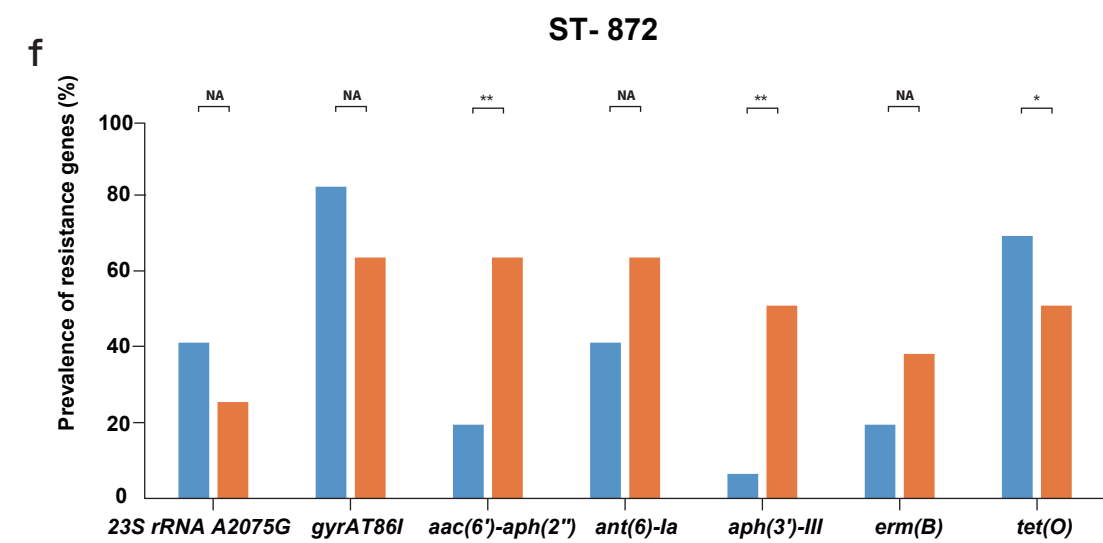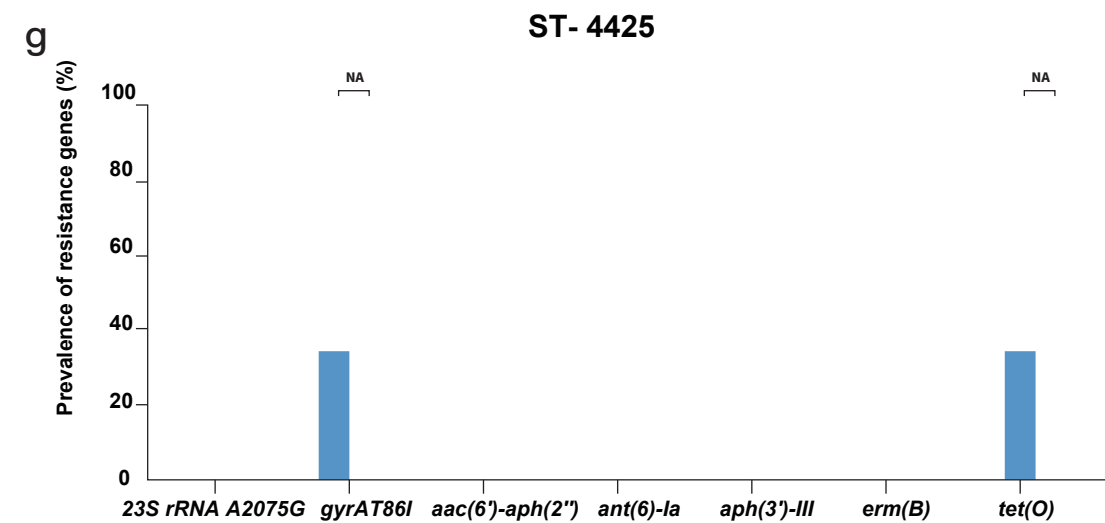

■ human  
■ poultry and associated environmental

Supplement: FIG S4 [file mbio.02835-22-s0004.pdf]

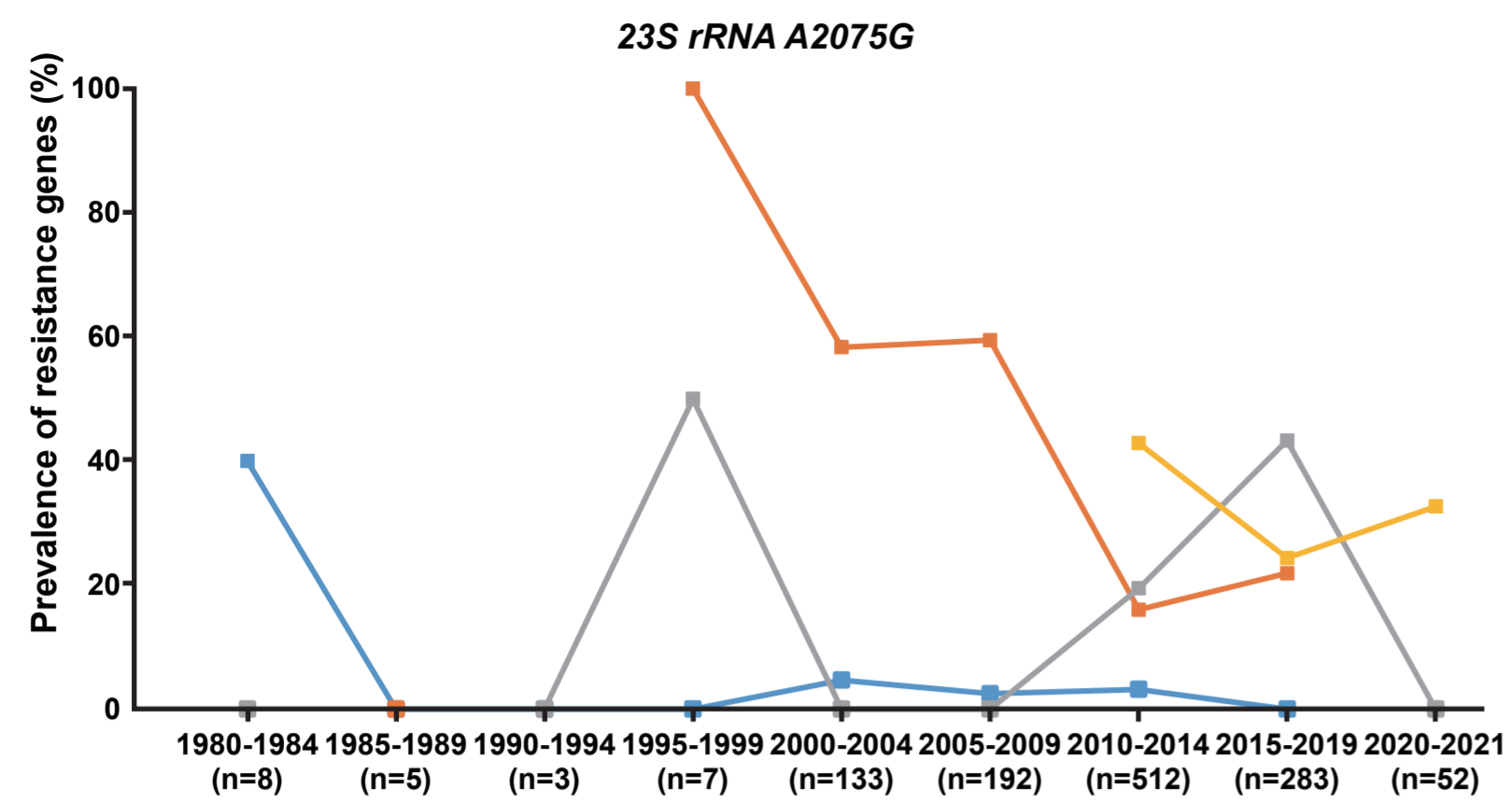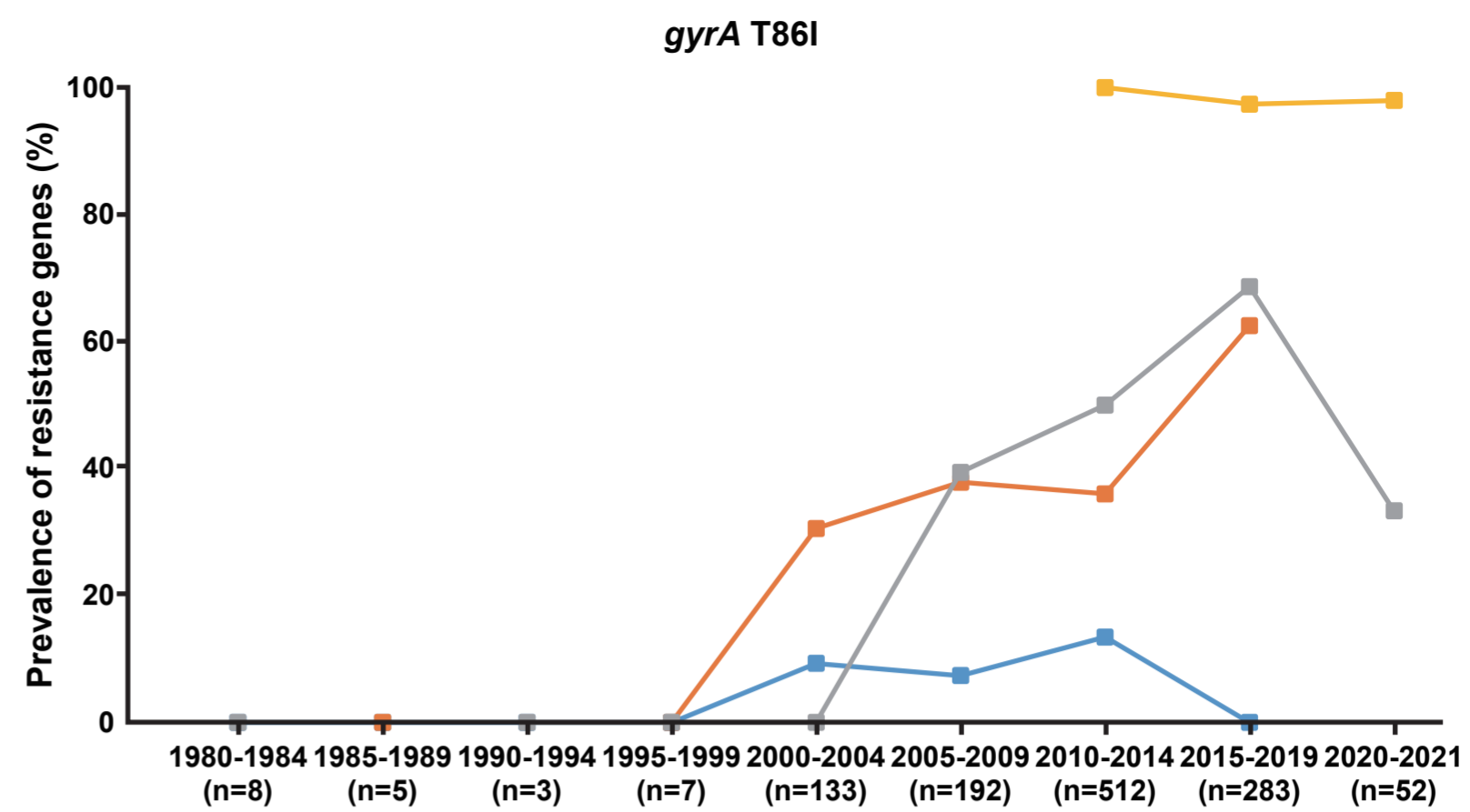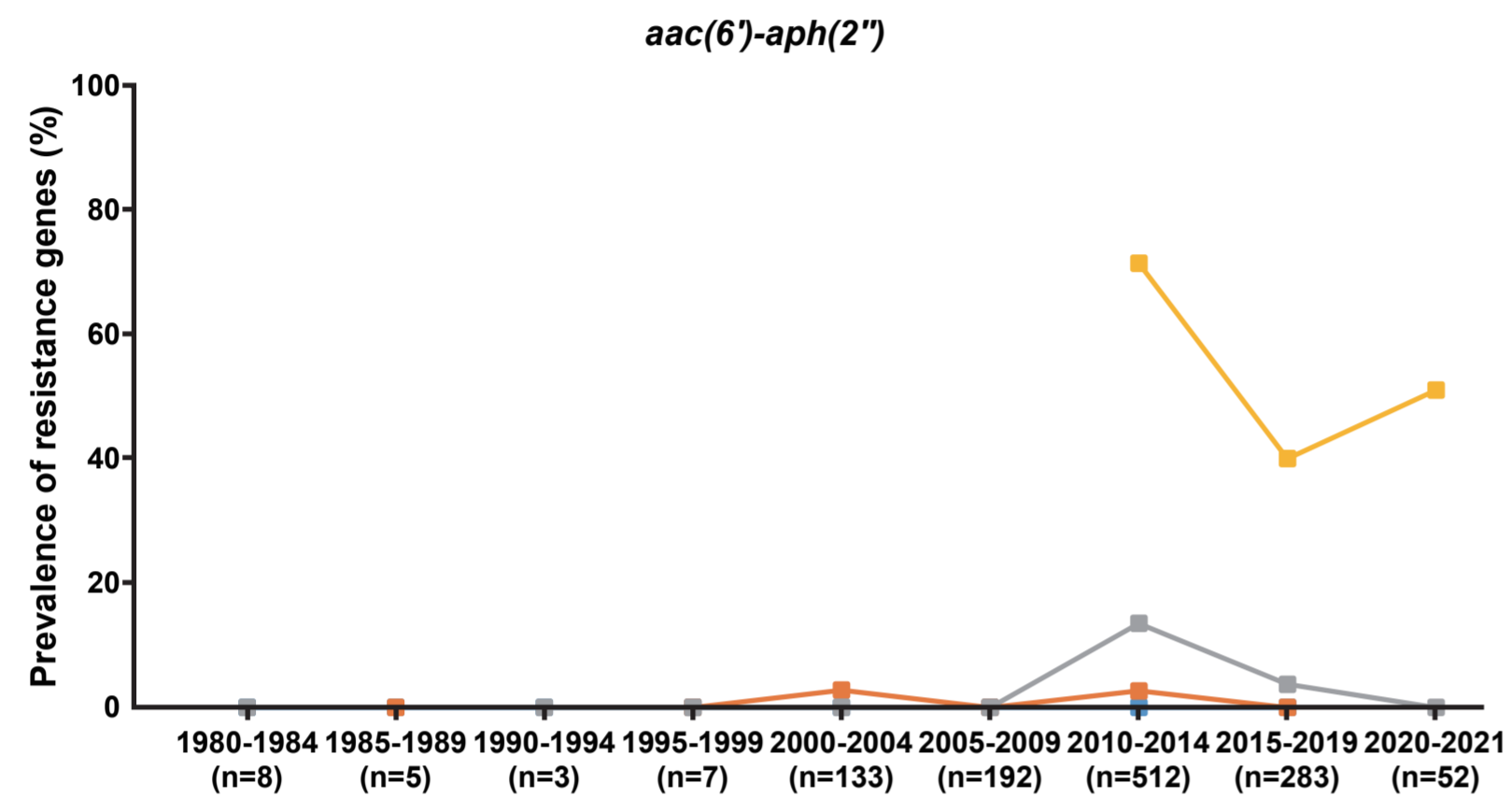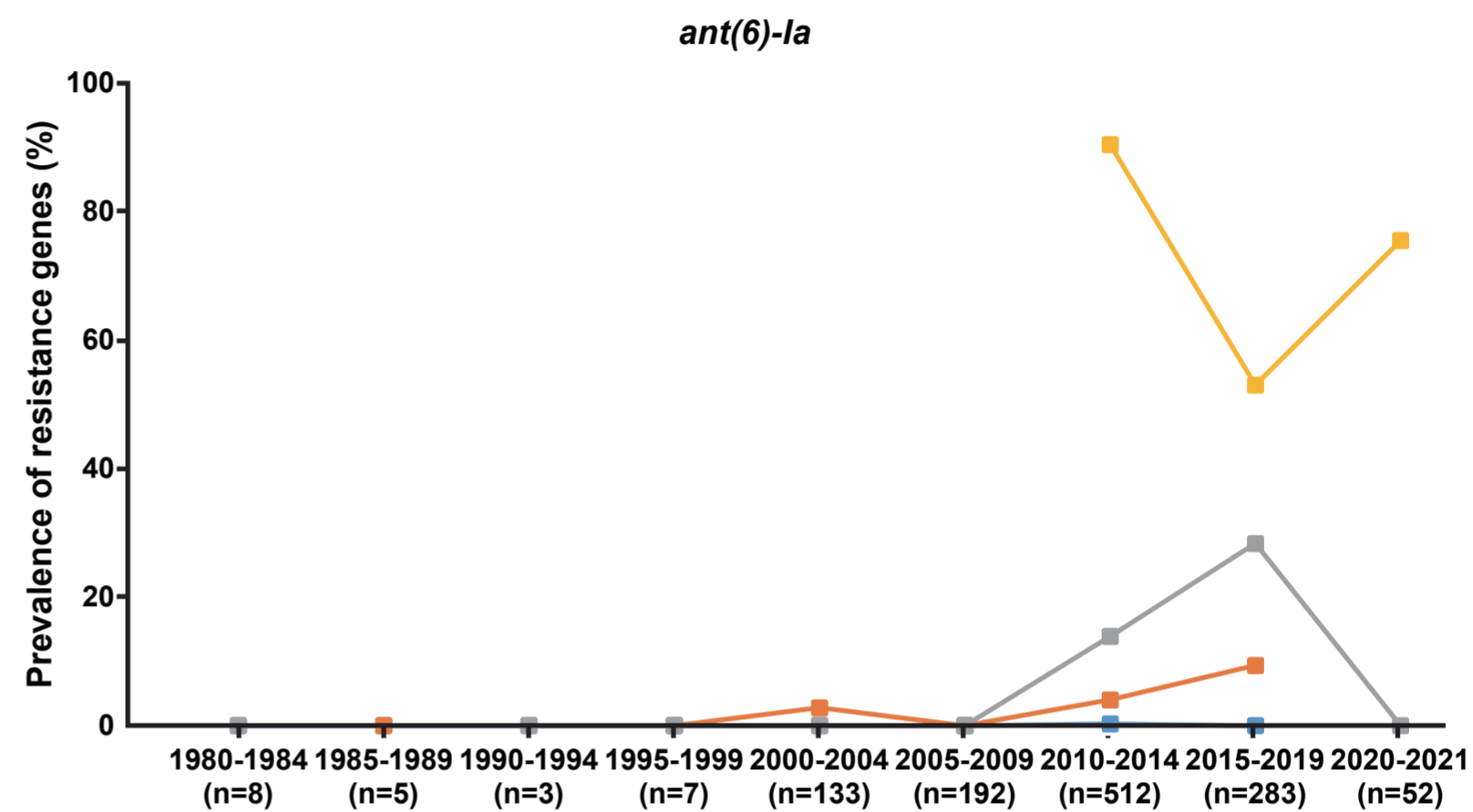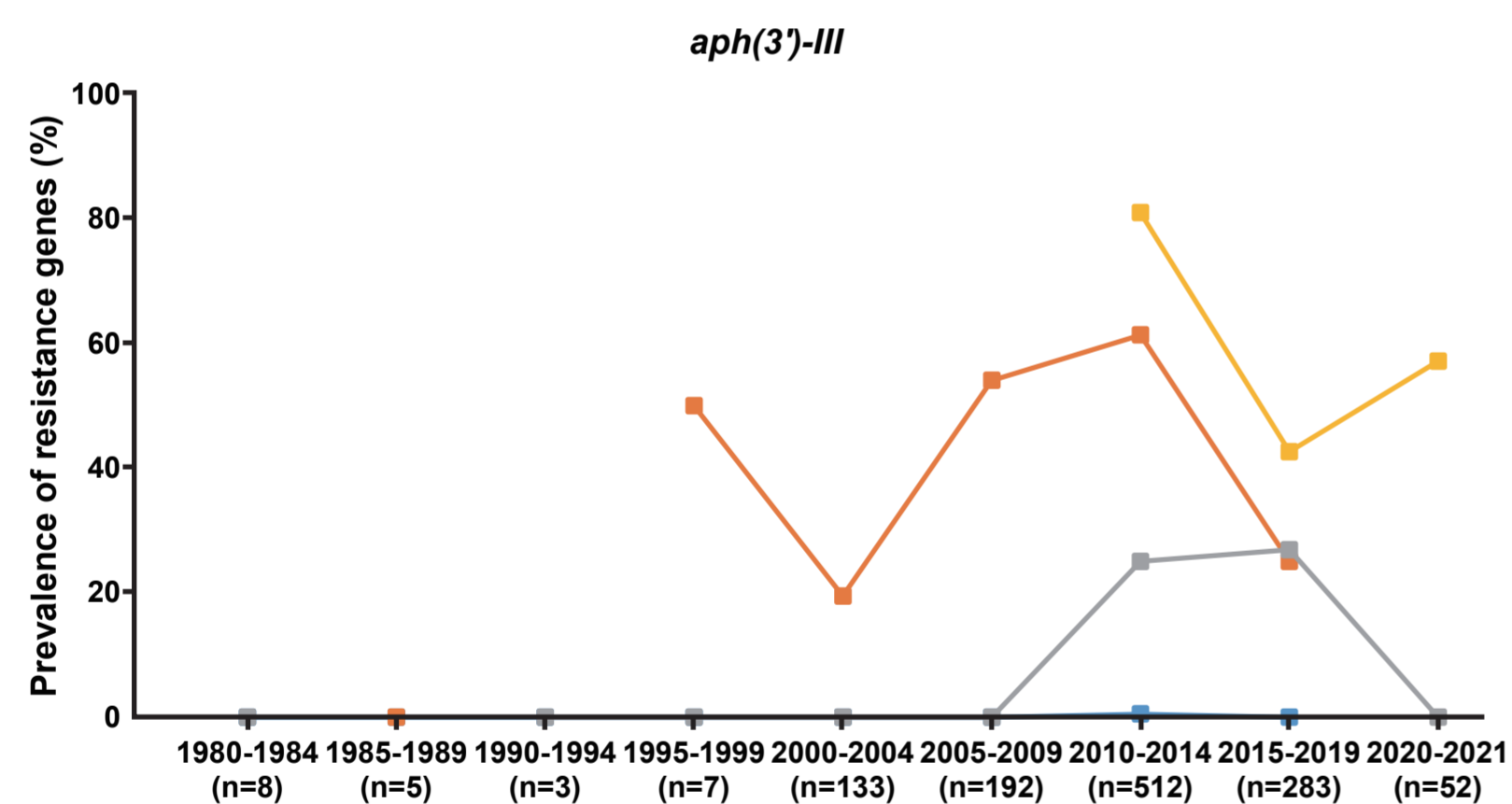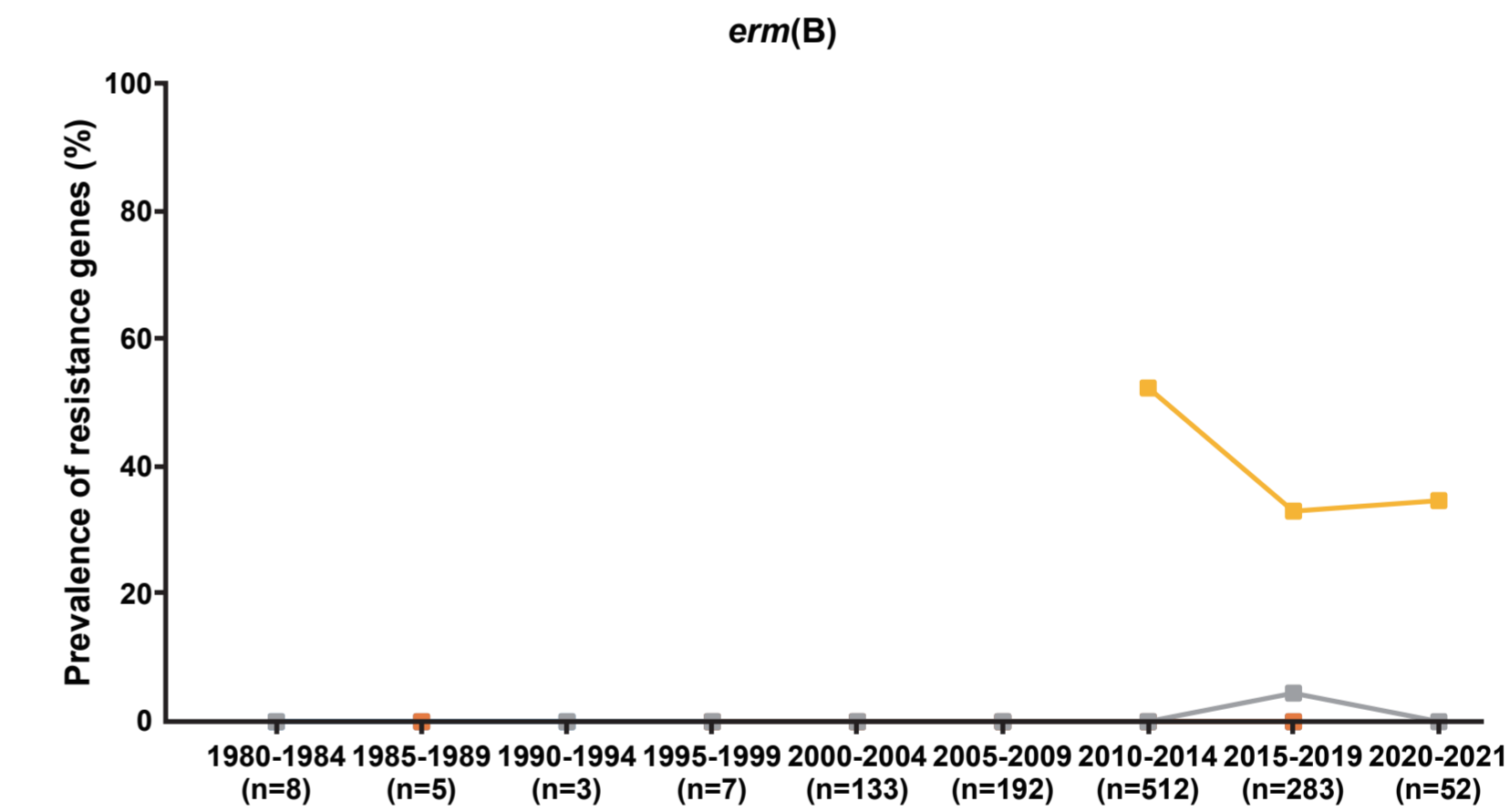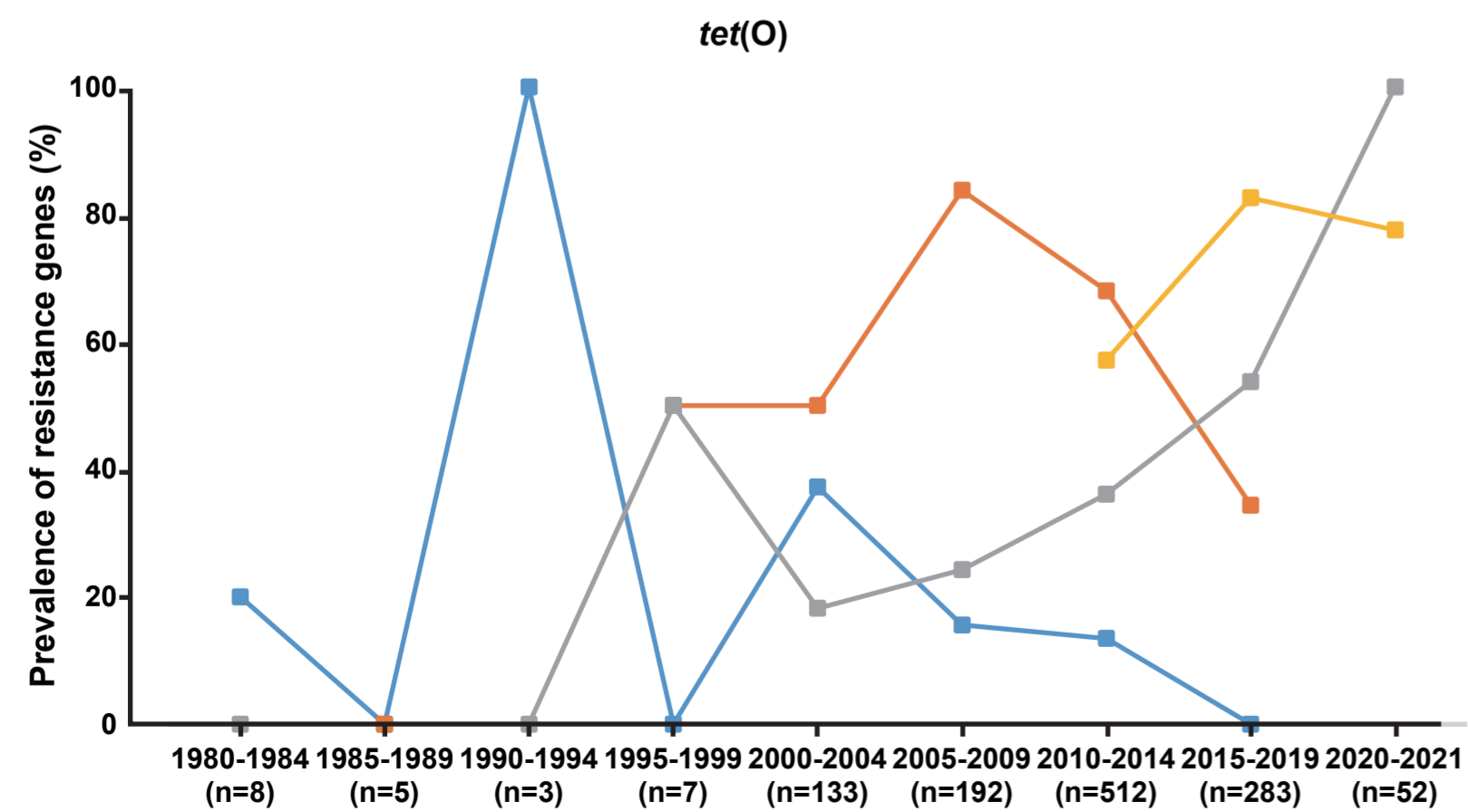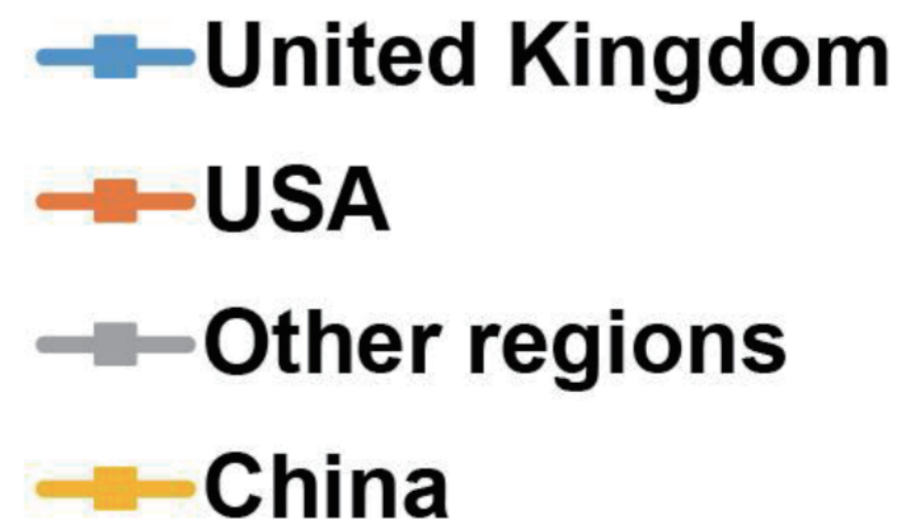

Supplement: FIG S5 [file mbio.02835-22-s0005.pdf]
